# Case report: hydroquinone and/or glutaraldehyde induced acute myeloid leukaemia?

**DOI:** 10.1186/1745-6673-1-19

**Published:** 2006-07-26

**Authors:** Vassilios Makropoulos, Evangelos C Alexopoulos

**Affiliations:** 1 Hellenic Institute for Occupational Health and Safety, Athens, Greece, Department of Occupational and Industrial Hygiene, National School of Public Health, Athens, Greece; 2 Occupational Health Department, Hellenic Shipyards SA, Athens, Greece, Department of Hygiene and Epidemiology, Medical School, University of Athens, Greece

## Abstract

**Background:**

Exposures to high doses of irradiation, to chemotherapy, benzene, petroleum products, paints, embalming fluids, ethylene oxide, herbicides, pesticides, and smoking have been associated with an increased risk of acute myelogenous leukemia (AML). Although there in no epidemiological evidence of relation between X-ray developer, fixer and replenisher liquids and AML, these included glutaraldehyde which has weakly associated with lymphocytic leukemia in rats and hydroquinone has been increasingly implicated in producing leukemia, causing DNA and chromosomal damage, inhibits topo-isomerase II, alter hematopoiesis and inhibit apoptosis of neoplastic cells.

**Case presentation:**

Two white females (A and B) hired in 1985 as medical radiation technologists in a primary care center, in Greece. In July 2001, woman A, 38-years-old, was diagnosed as having acute monocytic leukaemia (FAB M5). The patient did not respond to therapy and died threeweeks later. In August 2001, woman B, 35-year-old, was diagnosed with acute promyelocytic leukaemia (FAB M3). Since discharge, she is in continuous complete remission. Both women were non smokers without any medical history. Shortly after these incidents official inspectors and experts inspected workplace, examined equipment, archives of repairs, notes, interviewed and monitored employees. They concluded that shielding was inadequate for balcony's door but personal monitoring did not show any exceeding of TLV of 20 mSv yearly and cytogenetics analysis did not reveal findings considered to be characteristics of ionizing exposure. Equipment for developing photos had a long list of repairs, mainly leakages of liquids and increases of temperature. On several occasions the floor has been flooded especially during 1987–1993 and 1997–2001. Inspection confirmed a complete lack of ventilation and many spoiled medical x-ray films. Employees reported that an "osmic" level was continuously evident and frequently developed symptoms of respiratory irritation and dizziness.

**Conclusion:**

The findings support the hypothesis that the specific AML cases might have originated from exposure to chemicals, especially hydroquinone and/or glutaraldehyde. The report also emphasises the crucial role of inspection of facilities and enforcement of compliance with regulations in order to prevent similar incidents.

## Background

Most cases of acute myelogenous leukemia (AML) arise with no clear cause. Several factors have been associated with an increased risk of disease including exposures to high doses of irradiation, to chemical benzene, to chemotherapy etc. Anticancer drugs, mainly alkylating agents and topoisomerase II inhibitors, are the leading cause of treatment-associated AML. Inherited diseases with excessive chromatin fragility, e.g., Fanconi anemia, ataxia telangiectasia and, Kostmann syndrome, are associated with an increased risk of AML. Syndromes with somatic cell chromosome aneuploidy, e.g., Down (chromosome 21 trisomy), Klinefelter (XXY and variants), and Patau (chromosome 13 trisomy), are also associated with an increased risk of AML. In addition to benzene, exposure to petroleum products, paints, embalming fluids, ethylene oxide, herbicides, pesticides, and smoking are associated with an increased risk of AML.

Increased risk of leukaemia has been described among nursing and healthcare workers [1]. Radiologists and radiologic technologists comprise occupational groups exposed to low doses of ionizing radiation. To date, there is no clear evidence of increased leukaemia mortality in medical radiation workers exposed to current levels of radiation doses (below 20 mSv). Data from cohort studies of radiologic technologists provided statistically significant evidence of excess leukemia risk only among workers who were employed before 1950 eventhough recent cohorts of radiologic workers have not been followed up as long as the earlier ones [2-7].

Hydroquinone has been used in industry as a corrosion inhibitor, a fixative (graphics industry), substance of polystyrene manufacture, and in rubber production. Also it has been used for decades as a skin lightening agent but since January 2001 its use in cosmetics has been banned due to effects such as leukoderma-en-confetti or occupational vitiligo and exogenous ochronosis and concerns have raised regarding its carcinogenic potential [8,9]. Most of the evidence stems from research on benzene toxicity, which appears to arise via its metabolite hydroquinone. It causing DNA and chromosomal damage found in leukemia, inhibits topo-isomerase II, and alters hematopoiesis and clonal selection [10-14]. Additionally, hydroquinone is related to the inhibition of the apoptosis of neoplastic cells [15-17]. It is also hypothesized that background sources of hydroquinone and associated adducts, stem mainly from dietary ingestion, play a causal role in producing some forms of de novo leukemia in the general population [18]. On the other hand we have not up to now strong evidence and support of carcinogenicity from epidemiological studies with HQ and myelotoxicity associated with human exposure to HQ [19].

Glutaraldehyde is weakly associated with large granular lymphocytic leukemia in rats and has greater toxicity than formaldehyde [20].

Incidence of AML in white females in USA has risen to 3.6 per 100,000 people in 2001, the highest incidence of the period 1975–2003. Even though AML incidence increases dramatically among people who are over 40, a 20.5% of AML patients were diagnosed under age 44 during 1998–2002 in the United States compared to approximately 10.2% of patients of all types of cancers [21].

This study reports two cases of AML in workers potentially exposed to several chemicals and ionizing radiation.

## Case presentation

Two women (referred to as A and B in the text) were employed by Social Security Institute, the largest primary health care provider in Greece. They were hired in 1985, as X-ray assistants (radiographers) in a regional primary care center in a city of 40,000 inhabitants. Permanent personnel include also another two women, who were also employed by the regional center and hired at 1985 and 1993 without further referring in our report.

### Patient A

She was born at 1962; she was non smoker without any medical history. On the afternoon shift of 13 July 2001, she passed out and asked and received for a sick leave. Five days later, (18^th ^July 2001), she was admitted to haematology clinic of a big private hospital of Athens and diagnosed with acute myelogenous leukaemia. Bone marrow aspirate showed 65% blasts mainly (80%) with monocytic morphology. Flow cytometric immunophenotyping demonstrated expression of HLA-DR+/CD64+, CD36+/CD116+, CD14+/-, cCD68+, CD33++, CD13+/-, CD4+ and non expression of CD34, CD79a, CD20, CD10 and, CD3. Cytogenetic analysis showed a normal female karyotype of 46XX. These results were consistent with a diagnosis of acute monocytic leukemia (FAB M5). The patient did not respond to therapy and died three weeks later (6^th ^August 2001).

### Patient B

She was born at 1965, she was non smoker and from her medical history she had only a laparoscopic cholocystectomy in 1997. Her mother suffered from diabetes mellitus and coronary artery disease. She had never exposed to any other known factor predisposing for haematological disorder. In 13^th ^August 2001 she was admitted to Division of Hematology of University hospital of Athens, where she was hospitalized for eight months. Bone marrow aspirate showed 99% blasts. Blasts were of medium size with loose chromatin, thin cytoplasmatic granulations peroxidase-positive. Cytogenetics on the bone marrow sample showed a karyotype of 46, XX, t(15:17)(q22:q12). These results were consistent with the diagnosis of AML with t(15:17)(q22:q12), acute promyelocytic leukaemia according to the World Health Organisation classification (FAB M3). Since discharge from hospital on 23/3/2002 is in continuous complete remission.

### Occupational history and autopsies

Official inspectors from Ministry of Labour and medical and technical experts inspected workplace shortly after the incident (9–11/2001) and they examined equipment, archives of repairs, notes, and personal monitoring of employees [Additional files 1, 2, 3]. In addition employees were interviewed and based on that information it was concluded that:

The X-ray department of the Primary Care Center was first licensed in July 1986. The license should be renewed every 5 years. However, in the event that there is no radiologist in charge, as was the case during the periods 1987 to May 1994 and between April 2000 and August 2001, the license for the operation of the department should have been revoked. In July 1994 it was necessary to renew the license. However, further regulations stipulated shielding of the balcony door of the X-ray room and regular tuning or adjustment of the lamp current. In October 1995, the same preconditions were deemed necessary by the Greek Committee of Atomic Energy. The most important problem during the study period (1985–2001) seemed to be the absence of shield protection on the balcony door in the X-ray room. The female employees reported that due to a foul stench they often had to leave the X-ray room for the balcony. It is a matter of contention whether the employees were carrying the dosimeters at all times. If not the exposure would then be higher than the recorded one. In any case, the recorded exposure was ascertained from monthly records the period 1993–2001, never exceeded the safety limit levels of 20mSv prescribed by legislation.

The X-ray apparatus presented many faults in 1989 and for the following 2 years has presented problems with respect to high voltage readings and overheating. As a result, about 7500 films have been destroyed. The lamp current was adjusted only once in 1994. However, following a thorough check in June 1999 it was found that it was not possible to achieve high current intensities and adequate high voltages. It was suspected that current and voltage outputs were lower than the set values at high currents and voltages. The apparatus was used at the maximum dose rate of 4.5 R/min with a permissible upper limit equal to 5 R/min. In June 1999, it was also found that the filter in the X-ray lamp was 1.5 mm instead of 2.1 mm Al as required by legislation. It was replaced by a 3 mm Al filter. On the other hand, the apparatus could not achieve the correct currents and voltages and the maximum dose rate was lower than the expected.

Many serious problems were also associated with the development room. These included seepage from the tank, leakage from the waste faucet and an overheated working environment. From 1987 to 1992 over 20 problems associated with welding the tanks, short circuits, lack of thermostat control, seepage of waste and illegal disposal of wastes from fixing silver salts were recorded. During the period 1987–1990, all liquid wastes were collected in jerry cans, which often over spilled on the floor. The result was continues leakages of development and fixing liquids. This in conjunction with high temperatures and an inadequate exhaust system resulted in an unbearable stench and symptoms such as headaches, irritation of the eyes and the upper respiratory track and on occasion dyspnoea. The illegal disposal of wastes from fixing and developing salts compounded the problem.

The development apparatus (1985–1993) was replaced in 1993. However, new problems appeared in 1995, necessitating frequent alterations, which continued until 2001. In conjunction with a complete lack of proper ventilation, the stench and the symptoms presented by the employees persisted. All the above problems evident from the state and condition of the developing room, official notes and interviews are well documented. They now constitute proof of high exposure of the employees to toxic substances found in developing liquids. The chemical composition of the liquids is summarized in Table 1.

**Table 1:** Composition of X-ray developer, fixer and, replenisher liquids (TLVs based on Presidential Directive 90/1999)

INGREDIENTS	CAS NUMBER	Wt%	TLV-TWA*	TLV- STEL*
Acetic Acid	64-19-7	3–66	25 mg/m^3^ 10 ppm	37 mg/m^3^ 15 ppm
Aluminum Sulfate	10043-01-3	9,5		
Ammonium Thiosulfate	7783-18-8	40–60		
Boric acid	10043-35-3	1–5		
Diethylene Glycol	111-46-6	1–5		
Glutaraldehyde	111-30-8	30–40	0.8 mg/m^3^	0.8 mg/m^3^
Hydroquinone	123-31-9	6,2	2 mg/m^3^	4 mg/m^3^
1-phenyl-3-Pyrazolidinone	92-43-3	6,7		
Potassium Sulfite & metabisulfite	10117-38-1 & 16731-55-8	5–10		
Sodium Acetate	127-09-3	1–5		
Sodium Bromate	7789-38-0	0,5-1		
Sodium Sulfite	7757-83-7	5–10		
Sodium Tetraborate	1330-43-4	5–10	10 mg/m^3^	
Sulfuric acid	7664-93-9	4,6	1 mg/m^3^	
Water	7732-18-5	20–95		

*TLV-TWA: Threshold Limit Value-Time Weighted Average (8-hour workshift)

TLV-STEL: Threshold Limit Value-Short Term Exposure Limit (15-minute)

### Synopsis of aforementioned basic information

Three employees in an x-ray department worked under similar conditions for 16 years. Two of them were diagnosed in the period of a month as having acute myeloid leukaemia (AML). AML standardized incidence rate in women is approximately 1.5 cases per 100000 person-years in the age group of 35–39 years (14). In the small town, where the incident took place, the expected annual incidence of AML was less than 0.06 in the age group of 35–39 years. Consequently, the possibility of the reported incident to be random was extremely low. Since both employees had no medical history or exposure to any other known causal factor of leukaemia it was hypothesised that acute myelogenous leukaemia had an occupational origin.

**Figure 1:**
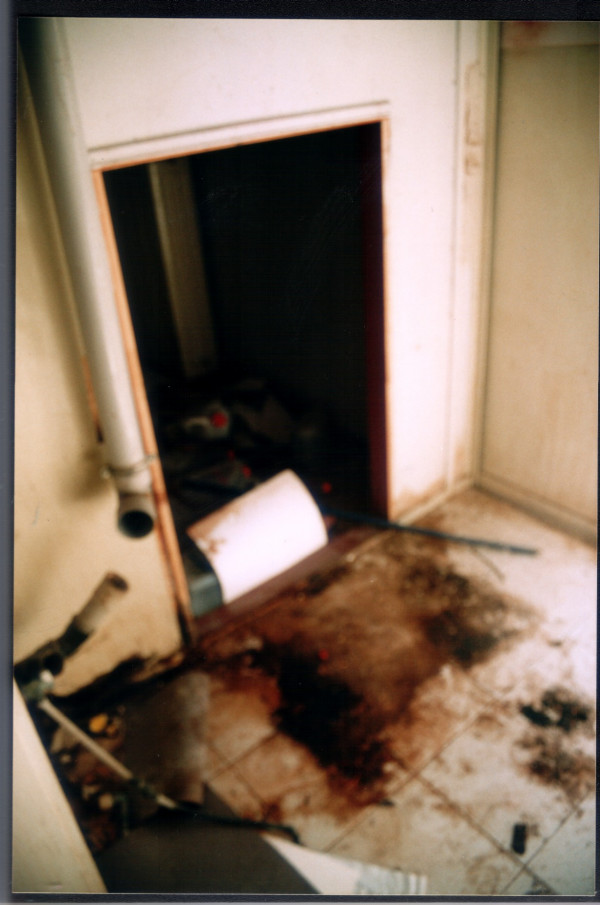
Stains from processing chemicals (photo of development room).

Experts' reports showed that: 1) the X-ray department had inadequate shielding at balcony's door 2) the X-ray apparatus could not achieve the correct currents and voltages and the maximum dose rate was lower than the expected, it presented many faults in 1989 and sporadically later, and the filter in the X-ray lamp was 1.5 mm instead of 2.1 mm Al. It was replaced by a 3 mm Al filter in June 1999 3) personal monitoring did not show any exceeding of TLV of 20 mSv yearly in film badges 4) extended cytogenetics analysis did reveal neither dicentric or polycentric chromosomes nor interstitial deletions, centric fragments or inversions considered to be characteristics of ionizing exposure 5) equipment for developing x-rays films had a long list of repairs, mainly leakages of liquids and increases of temperature. Many times floor has been flooded especially during 1987–1993 and 1997–2001. Floor had brown stains form effluent chemicals [Figure 1]; 6) in the development apparatus room there was complete lack of ventilation and many spoiled medical x-ray films 7) employees reported that an "osmic" level was continuously evident and frequently developed symptoms of headache, respiratory irritation and dizziness. These reports confirmed from notes of employees protesting about working conditions and in addition there was not any medical file of employees and health monitoring.

Data collected from various sources like autopsies in workplace and inspections of equipment and archives of official notes; employees' interviews; film badges etc. provided strong evidence that employees were exposed constantly to harmful X-ray developer, fixer and replenisher liquids over a 16-years period. These substances included hydroquinone, glutaraldeyde, acetic acid, 1-phenyl-3-pyrazolidone, sodium sulphite, sulphuric acid and other chemicals. Glutaraldehyde has weakly associated with large granular lymphocytic leukemia in rats while hydroquinone (HQ) has been increasingly implicated in producing leukemia. In addition sulfuric acid mist correlated with lung, nasal and larynx cancers. Even though it is well documented that HQ in the effluent from photo processing and wastewaters had been present for a long period, HQ should not volatilize easily because of its water solubility, very low vapour pressure, and high vapour density. HQ, in an open body of water, would be expected to both biodegrades and photo degrades. In the presence of moisture and ambient level of oxygen, hydroquinone undergo oxidation to 1,4-benzoquinone, which is more likely to volatilize because of its higher vapour pressure [22]. High temperatures had enhanced this confirming by brown stains near central heating mode. So it is anticipated that exposure of employees via inhalation include both hydroquinone and 1,4-benzoquinone. In addition exposure through skin might be happened in a lesser extent.

There is always, a small possibility, for employees to be exposed to radiation. This is limited to the hypothesis that employees spent time in balcony, where shielding of the door was not sufficient (adequate). This habit might contribute to radiation exposure in case those employees did not wear their film badges. In contrast was that extended cytogenetics analysis did not reveal either dicentric or polycentric chromosomes or interstitial deletions, centric fragments or inversions considered to be characteristics of ionizing exposure. Radiation and hydroquinone could induce a chromosomal instability that may contribute to AML development by increasing the number of genetic lesions in hemopoietic cells. Recent research showed that this effect could be induced by hydroquinone doses that are not acutely stem cell toxic [23]. Preliminary findings showed a synergistic effect of hydroquinone when combined with ionizing radiation in terms of SCEs, and a possible synergistic effect when chromatid breaks are analysed after G2-phase irradiation [24].

Radiographers or medical radiation technologists are potentially exposed to multiple chemicals in processing and developing radiographs, which can cause irritation of the eyes and upper airways, asthma-like symptoms (cough, wheeze or dyspnea), headaches, dizziness, fatigue, etc. "Darkroom disease" (DRD) has been used to describe unexplained multiple symptoms attributed by radiographers to their work environment. Exposure to chemicals may occur through leaks and from mists and vapors from the processor, from film and through pouring chemicals. Inadequate ventilation, frequently detecting odor of X-ray processing chemicals and cleaning up spills were highly associated with most of the symptoms. It is possible that acetic acid, sulphur dioxide, or other volatile chemicals such as glutaraldehyde might account for irritant and/or odorrelated nasal, eye, and other irritant effects [25-31].

Hydroquinone is odourless but alkaline solutions readily form 1,4-benzoquinone which has odour threshold of 0.1 ppm. Glutaraldehyde has an odor threshold of 0.04 ppm (0.16 mg/m^3^) in air, acetic acid has an odor threshold ranging from 2.5 to 250 mg/m^3^, and that for sulphur dioxide ranges from 1.18 to 12.5 mg/m^3 ^but measured levels of radiographers' exposure to glutaraldehyde, acetic acid, and sulfur dioxide were not associated with reported odor. Because measured exposures to glutaraldehyde were considerably lower than this, glutaraldehyde is less likely to have been detected by the study subjects.

Surveys have shown that x-ray workers are exposed to glutaraldehyde levels between 1–10 μg/m^3^, and for acetic acid, and sulphur dioxide less than 0.1 mg/m^3 ^[29-33]. Concentrations of other chemical constituents as well as their interactions are not well known. Under routine conditions, Teschke et al., found that exposures to X-ray processing chemicals have been shown to be well below the levels permitted by most regulatory agencies [30]. Airborne concentrations of glutaraldehyde measured after a spill of 2% activated solution were found to greatly exceed exposure limits [34].

## Conclusion

To our knowledge, the cases presented here are the first reported cases of AML probably related to exposure to X-ray developer, fixer and, replenisher liquids. Hydroquinone and in a lesser degree glutaraldehhyde are hypothesised to be the causal factors. Perhaps combined exposure to other chemicals or to low dose x-ray might have played a role.

In this facility an average of 300 films per week processed and the consumption of developer (part A-hydroquinone 6.2%) was estimated around 7.5 lt. The complete lack of direct local exhaust ventilation of the processing machine (and insufficient general room ventilation), the excessive radiographic film processor heating, the high room temperature (30–35°C), and the irregular or no use of silver recovery unit could increase volatility of chemicals including hydroquinone. In addition, personnel besides regular duties (taking X-rays, waiting in the processing area, refilling chemicals and observing film) had cleaned up spills several times without using any personal protective equipment (gloves and goggles). Based on these circumstances, we anticipated levels of exposure well above permitted levels (Table 1). In addition few tasks with potential for skin-wetting exposures have frequently taken place (e.g., cleaning the processor, refilling chemicals or cleaning spills) so some dermal exposure might contributed to higher exposure levels.

For hydroquinone, only recently, has been defined an occupational exposure limit in Greece (PD 90/7.5.1999) in addition to the fact that any kind of environmental monitoring is highly unexpected in regional public health sector. The latter emphasises the need for preventive actions such as information for employees about the possible toxic effects of chemicals and education on proper handling, storing and, disposal. In our case it seems that adequate ventilation might have been sufficient for personal protection. For most of the chemical factors as clearly was indicated in material safety data sheets (MSDS) for exposure control and personal protection it was recommended to ventilate work area, with ten (10) or more air changes per hour. Under normal operating conditions and with adequate ventilation and proper storage the exposure would be minimized. The failure to protect the employees seemed to be due to failure to comply with existing occupational safety and health rules. This also emphasises the significance of inspection of facilities and enforcement of compliance with regulations in order to prevent similar incidents.

## Competing interests

The author(s) declare that they have no competing interests.

## Authors' contributions

VM and ECA have been involved in analysis, interpretation of data and, in drafting the manuscript. Both approved the final manuscript.

## Supplementary Material

Additional File 1 Archive of repairs of developing apparatus. The data provided represent the archive of repairs of developing apparatus the period 1985–2001. Click here for file

Additional File 2 Official administrative notes of employees to employer. The data provided represent the notes/requests of employees the period 1990–2000. Click here for file

Additional File 3 District attorney's report. The data provided represent selected text from district attorney's report. Click here for file
