# Supplementary figures and images for: Case report: hydroquinone and/or glutaraldehyde induced acute myeloid leukaemia?
Source: J Occup Med Toxicol. 2006 Jul 26;1:19. doi: 10.1186/1745-6673-1-19 (PMC1544343; doi:10.1186/1745-6673-1-19)

| 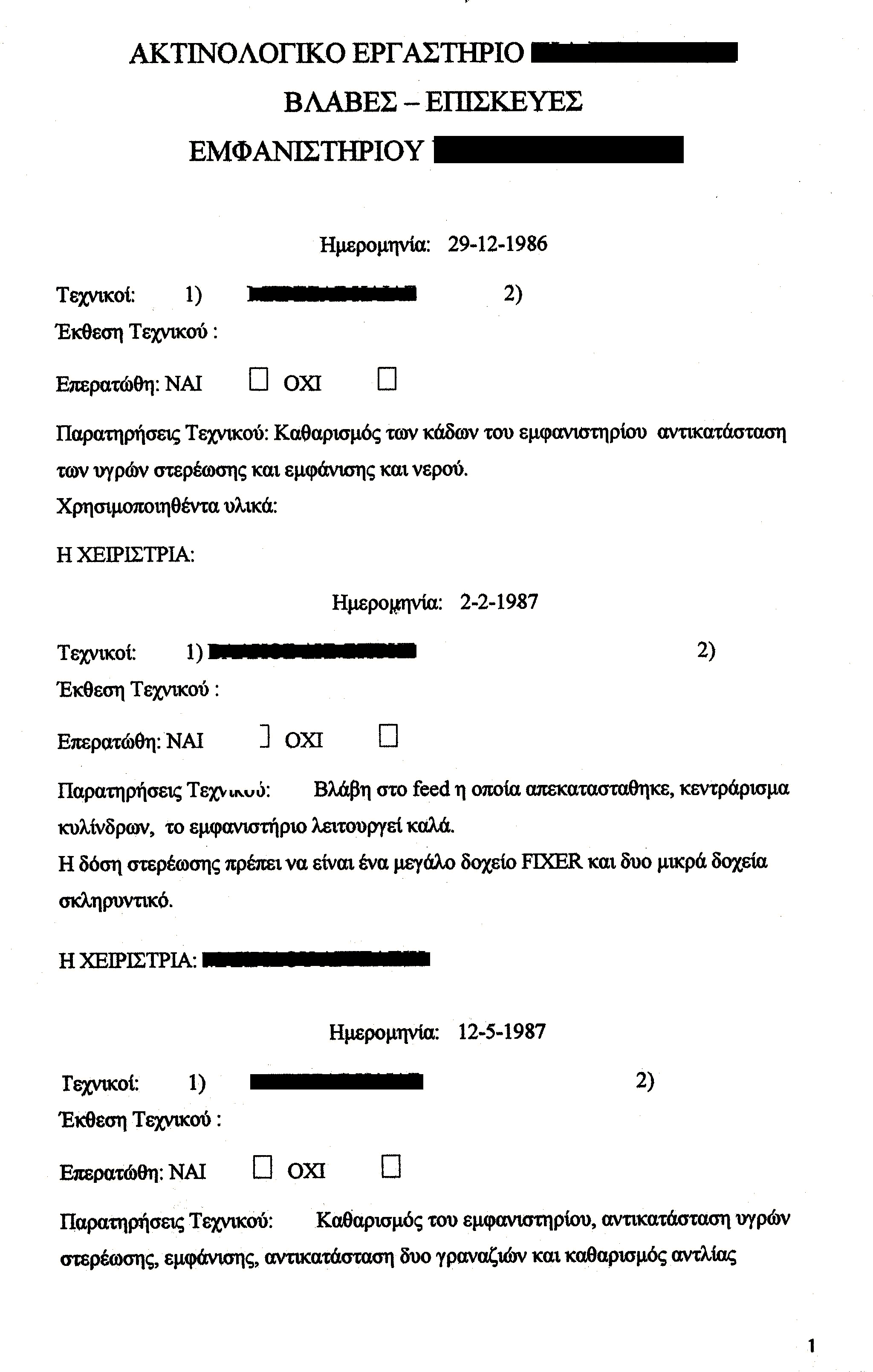 | 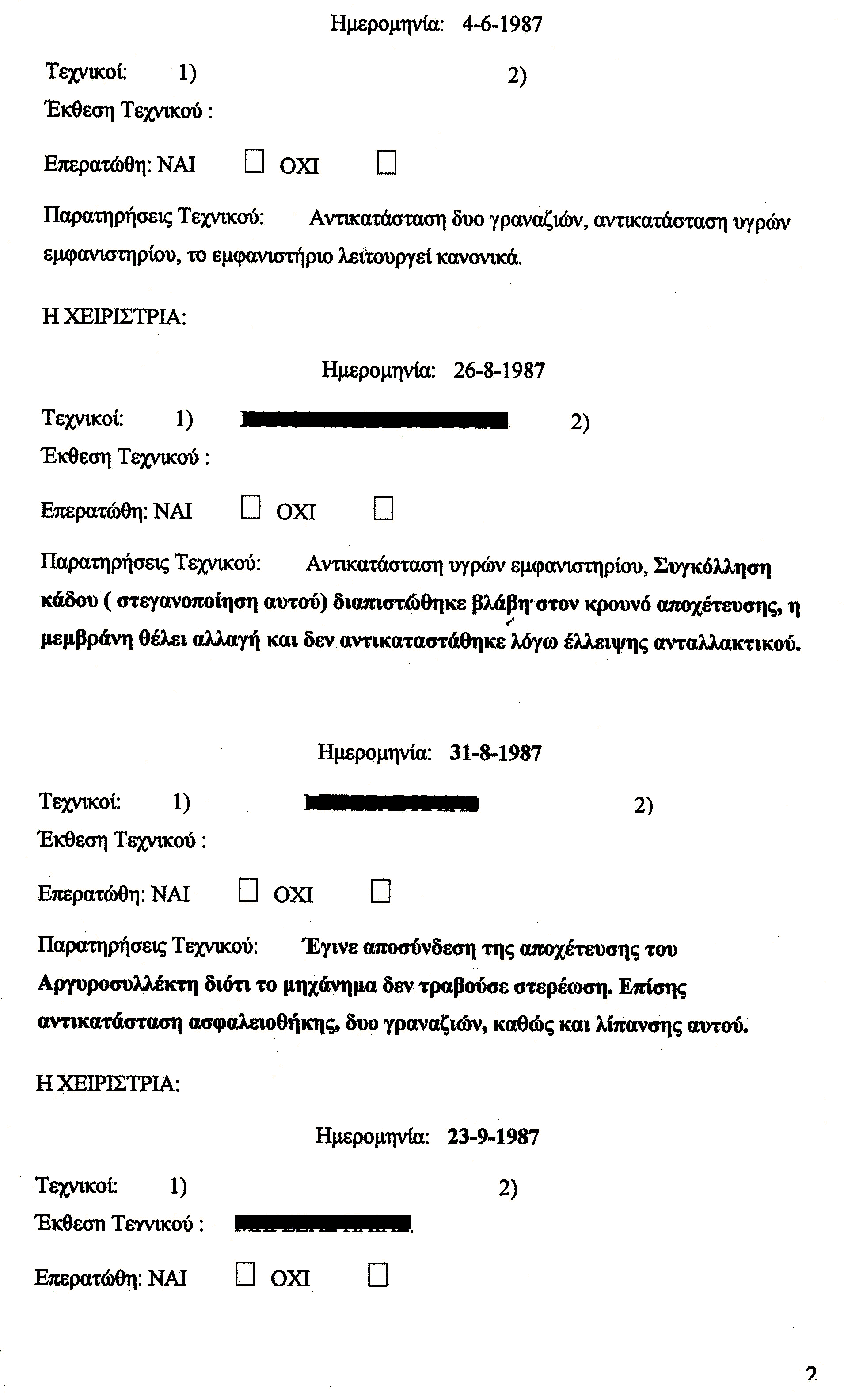 |
| --- | --- |

| 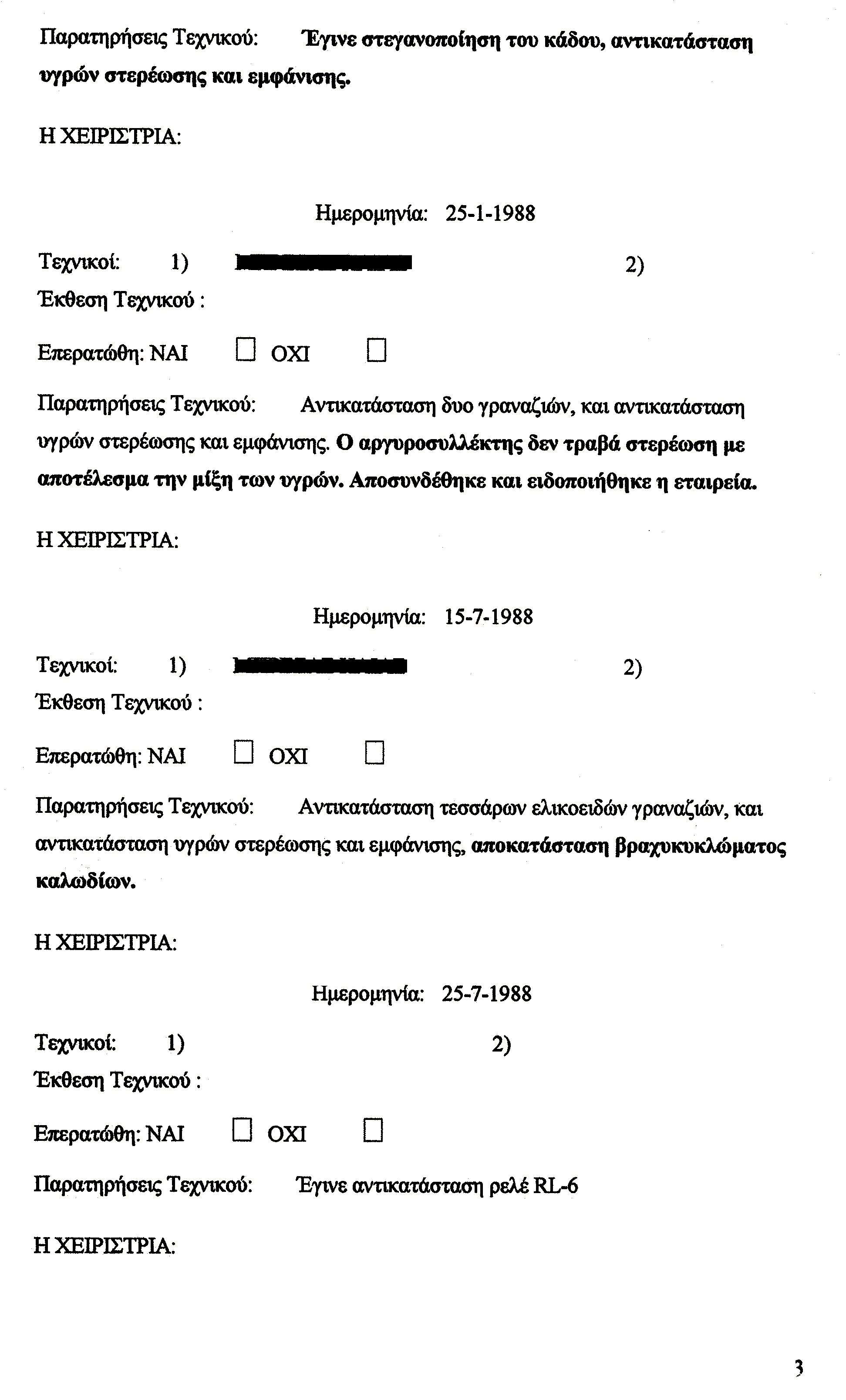 | 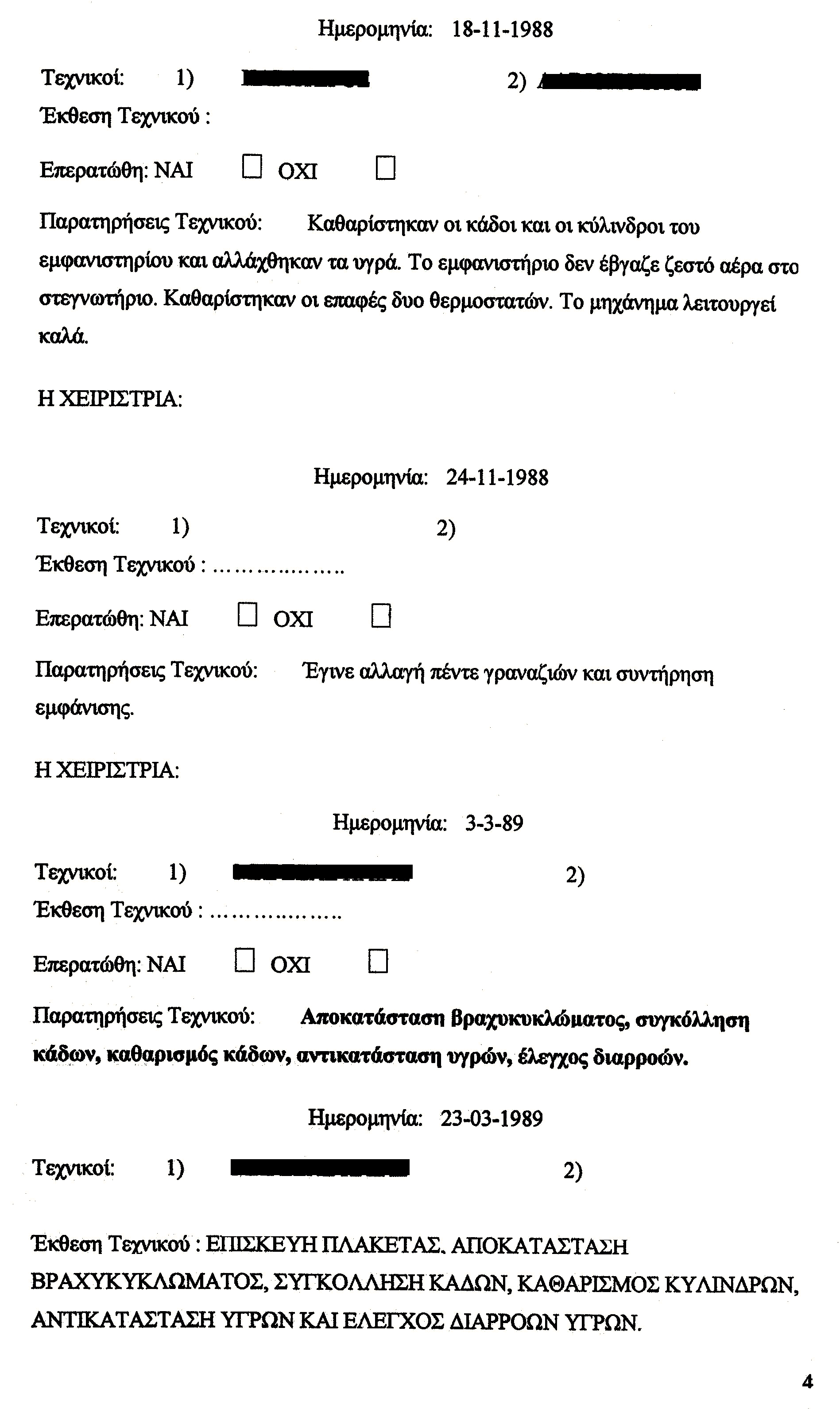 | |
| --- | --- | --- |
| 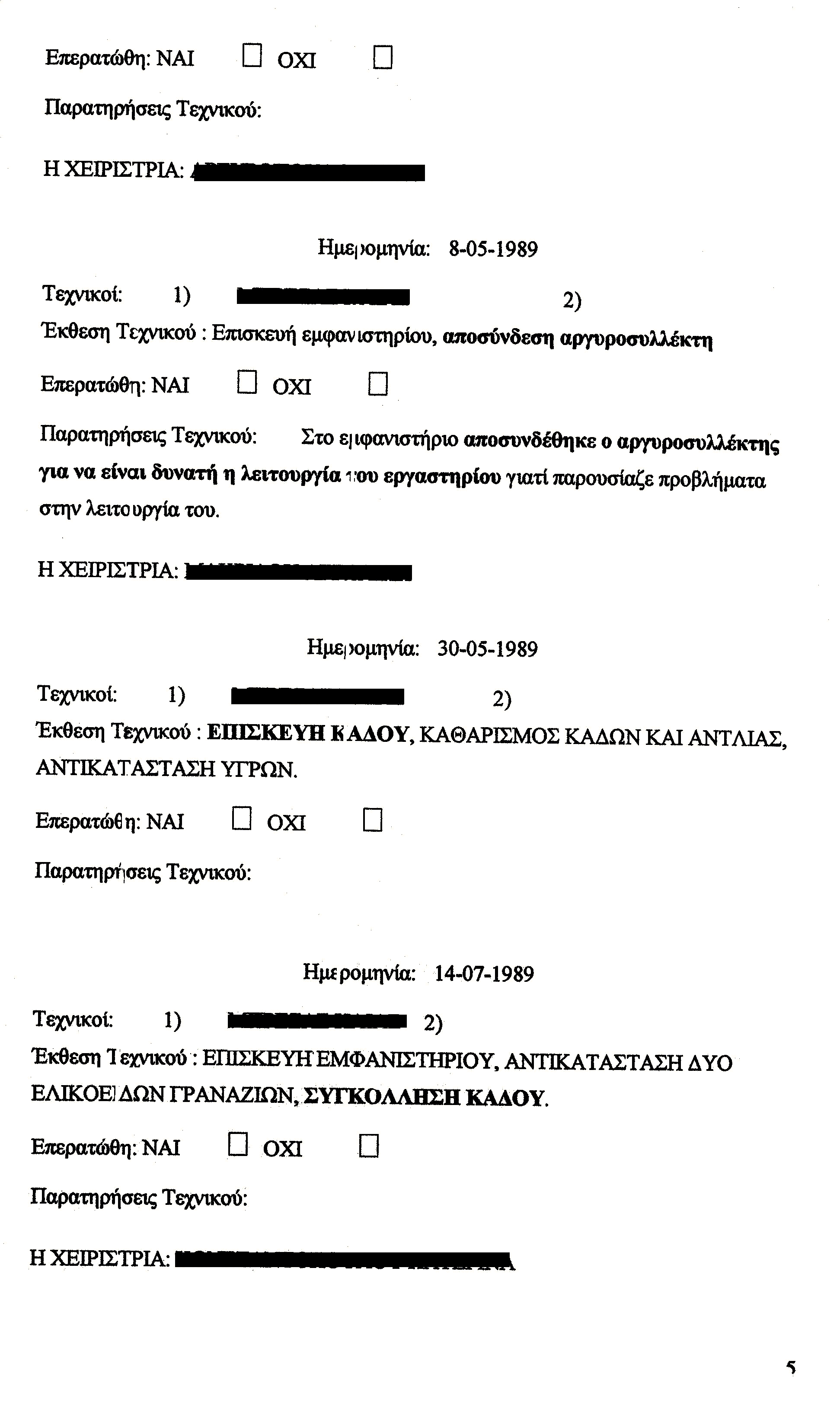 | 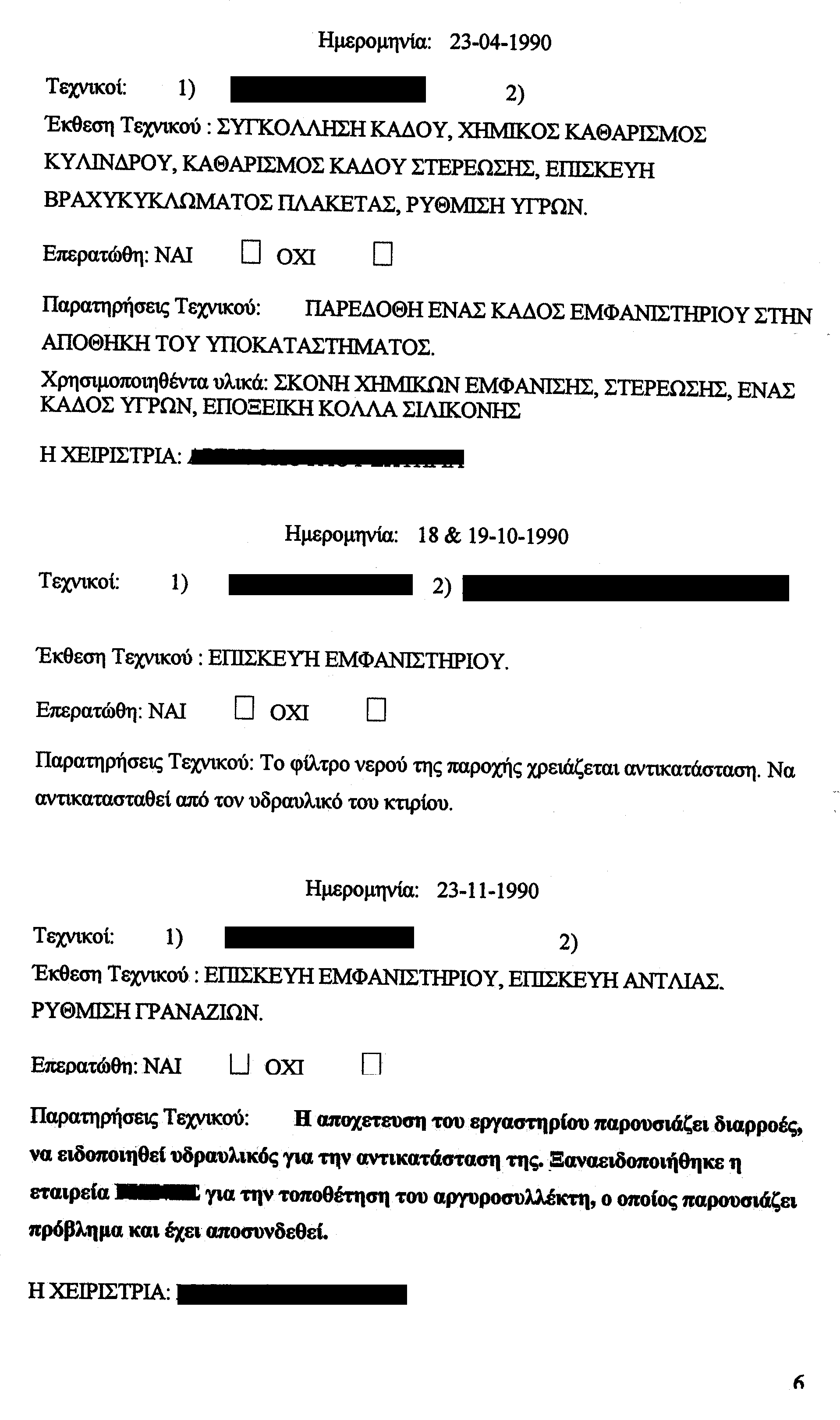 |  |

| 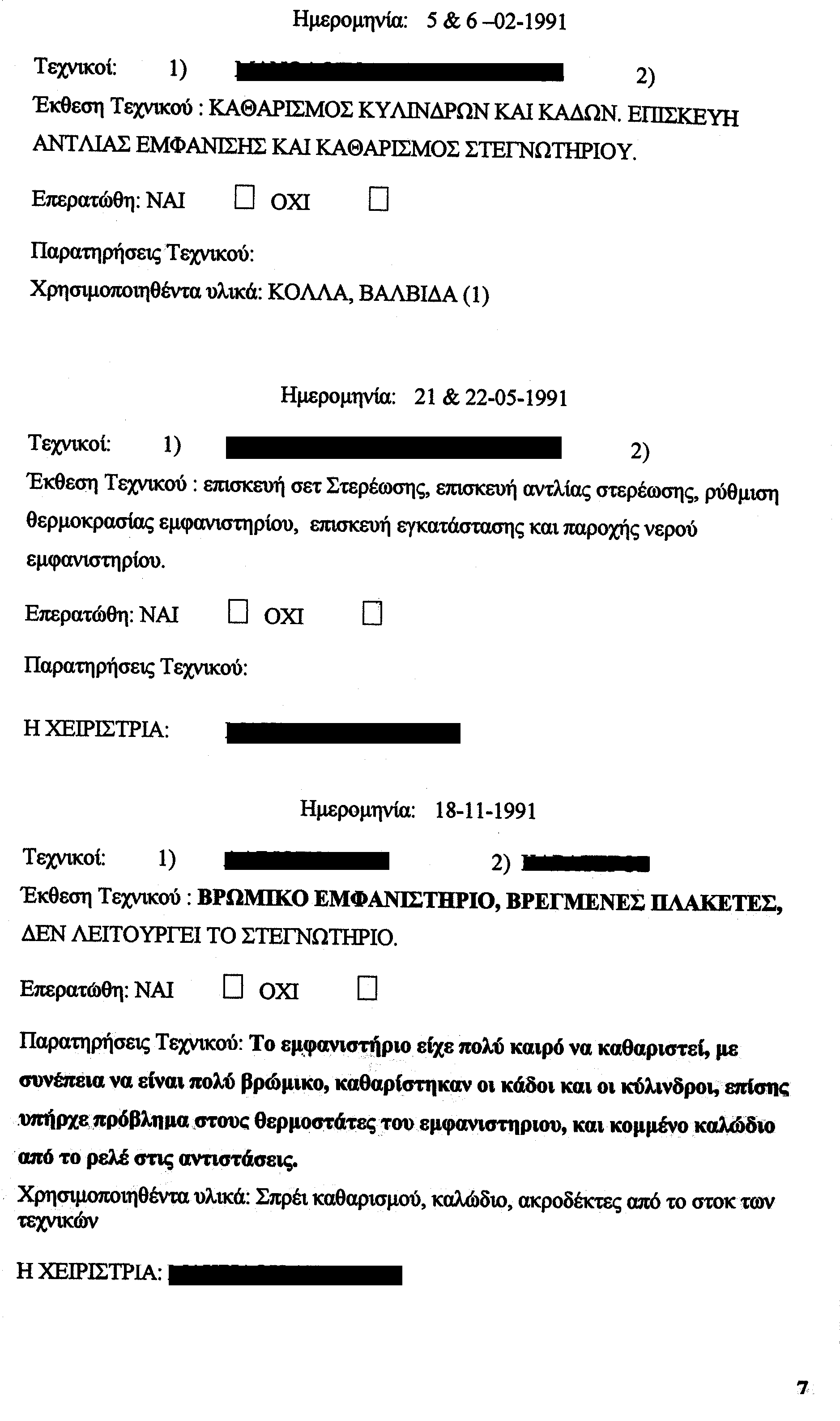 | 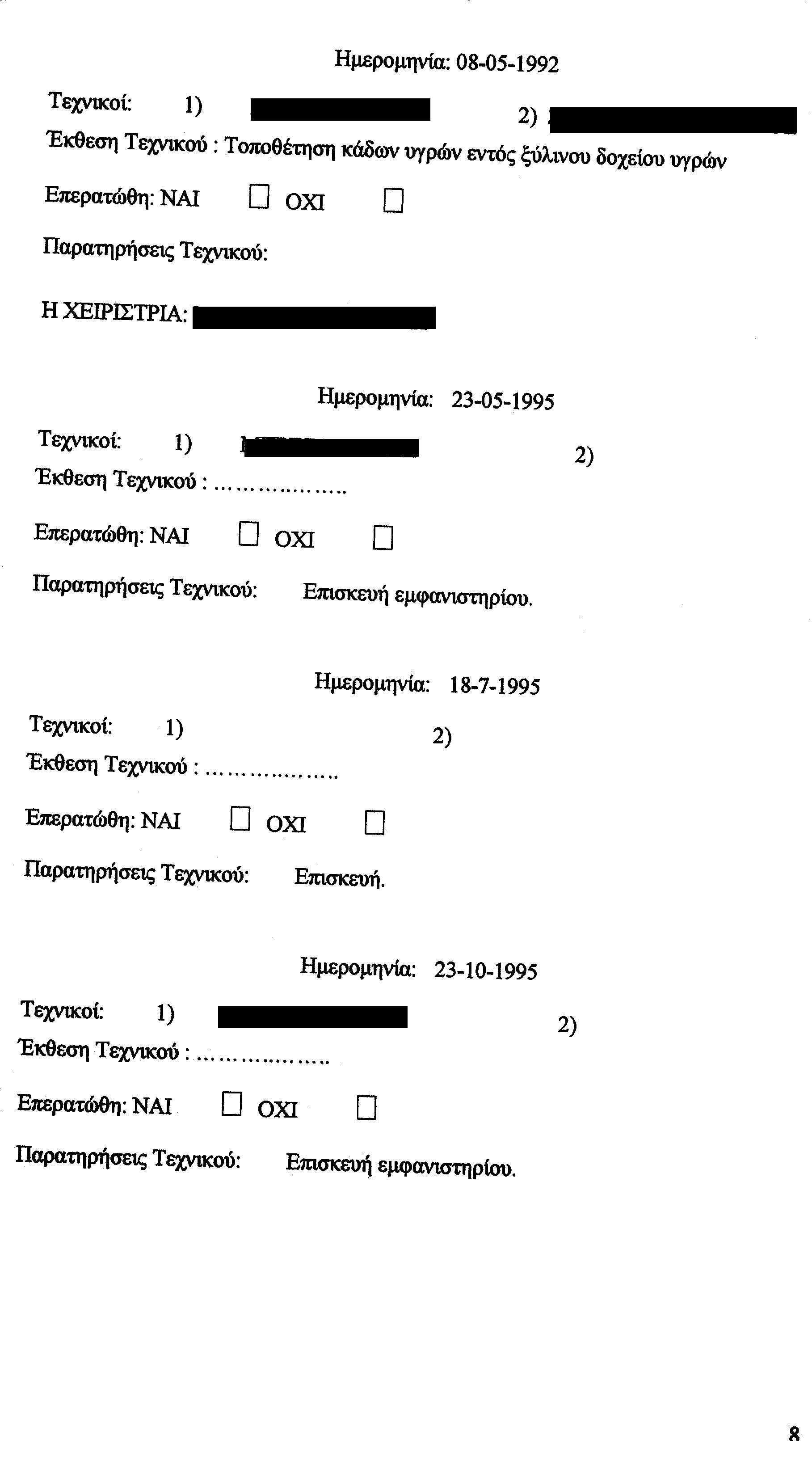 |
| --- | --- |

| 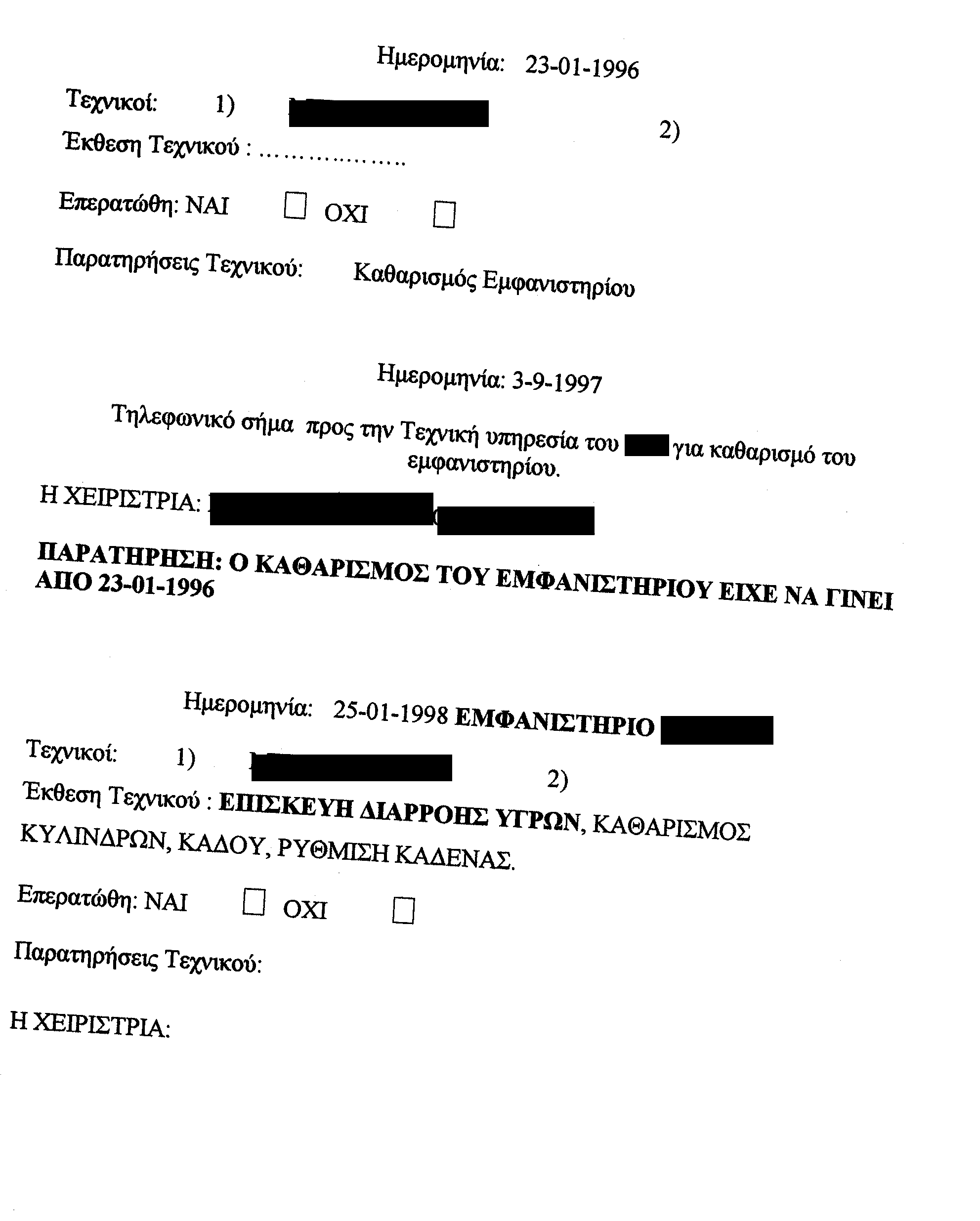 |  |
| --- | --- |

Supplement: Additional File 1 — Archive of repairs of developing apparatus. The data provided represent the archive of repairs of developing apparatus the period 1985–2001. [file 1745-6673-1-19-S1.doc]

| 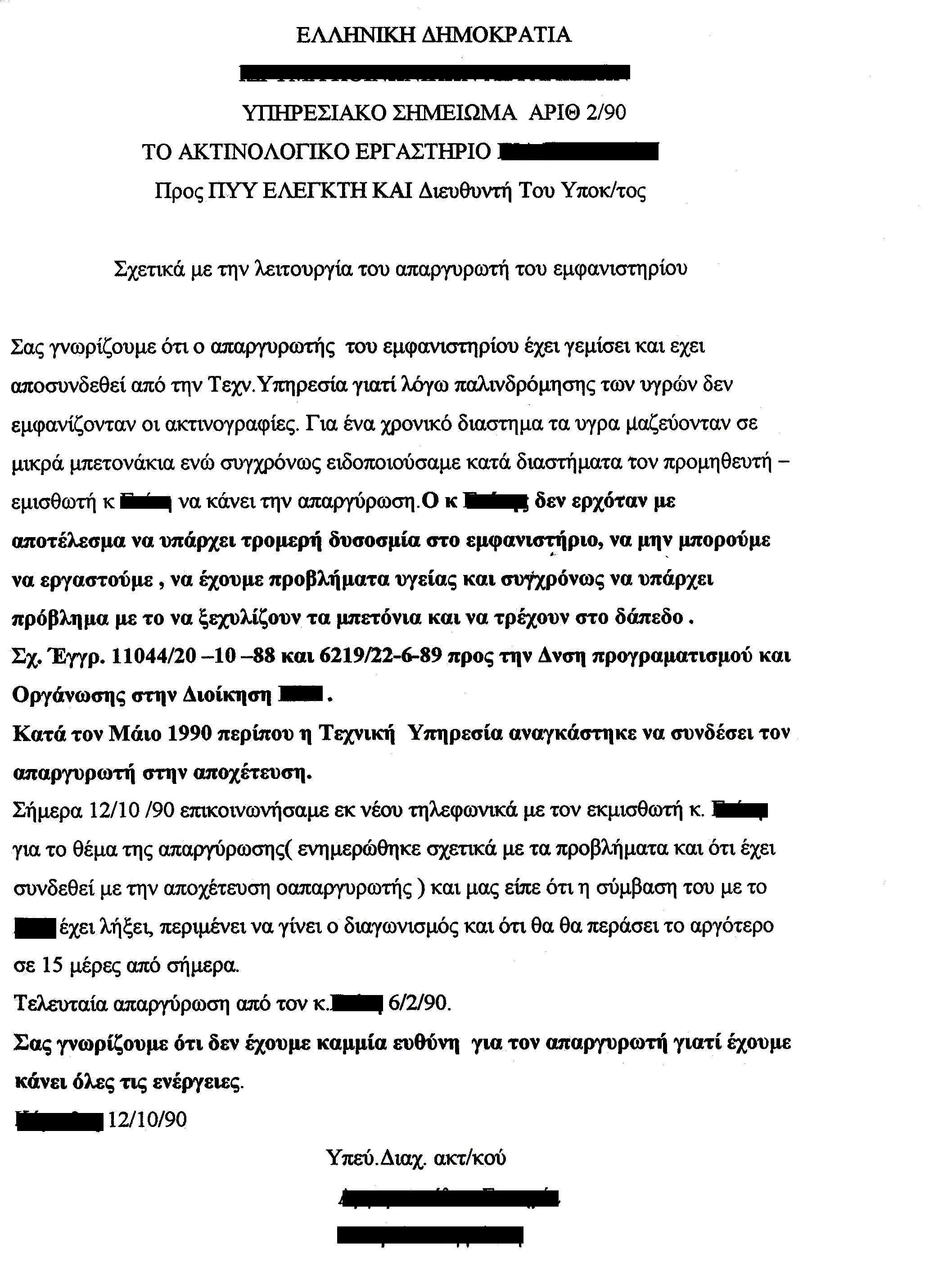 | 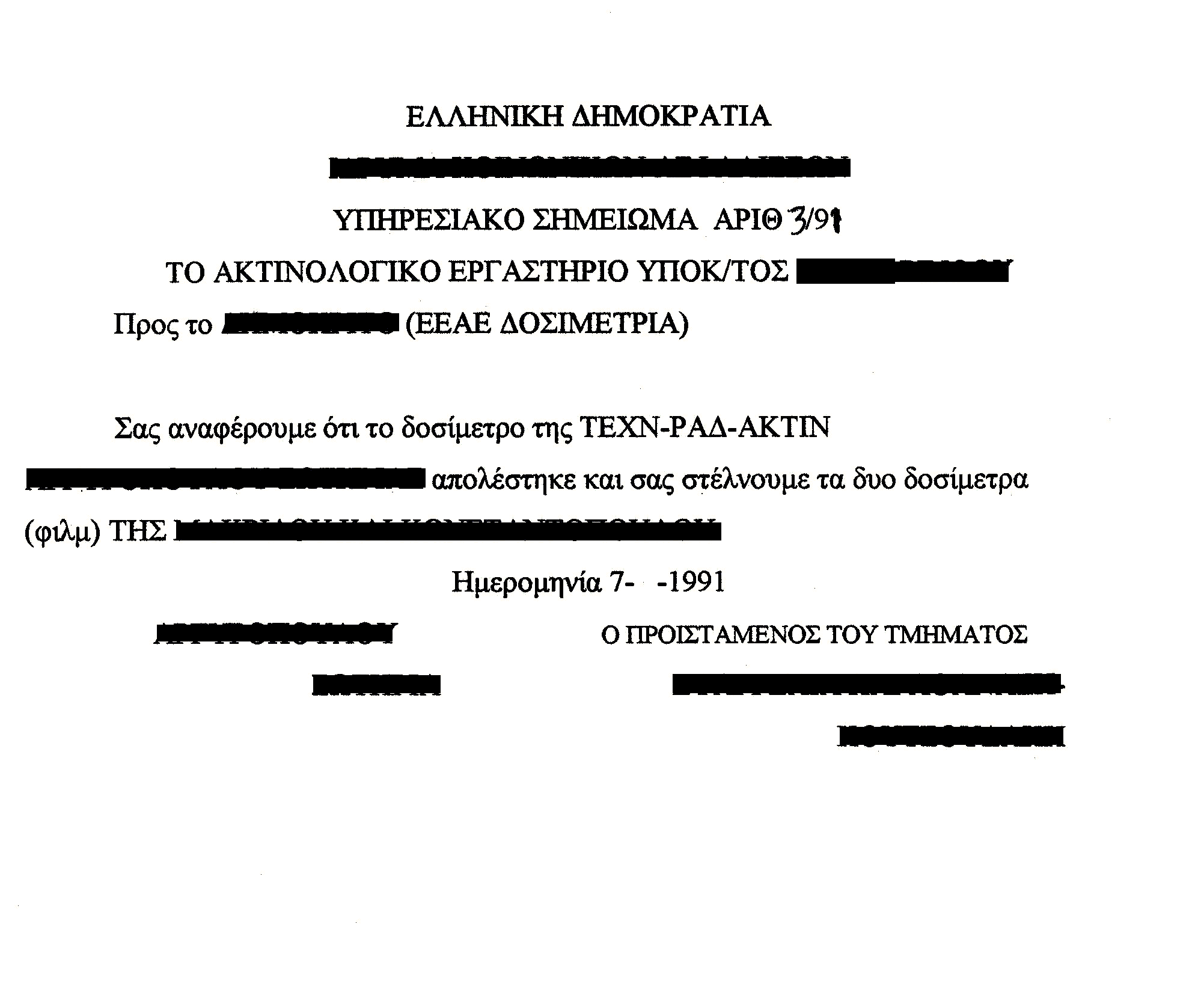 |
| --- | --- |

| 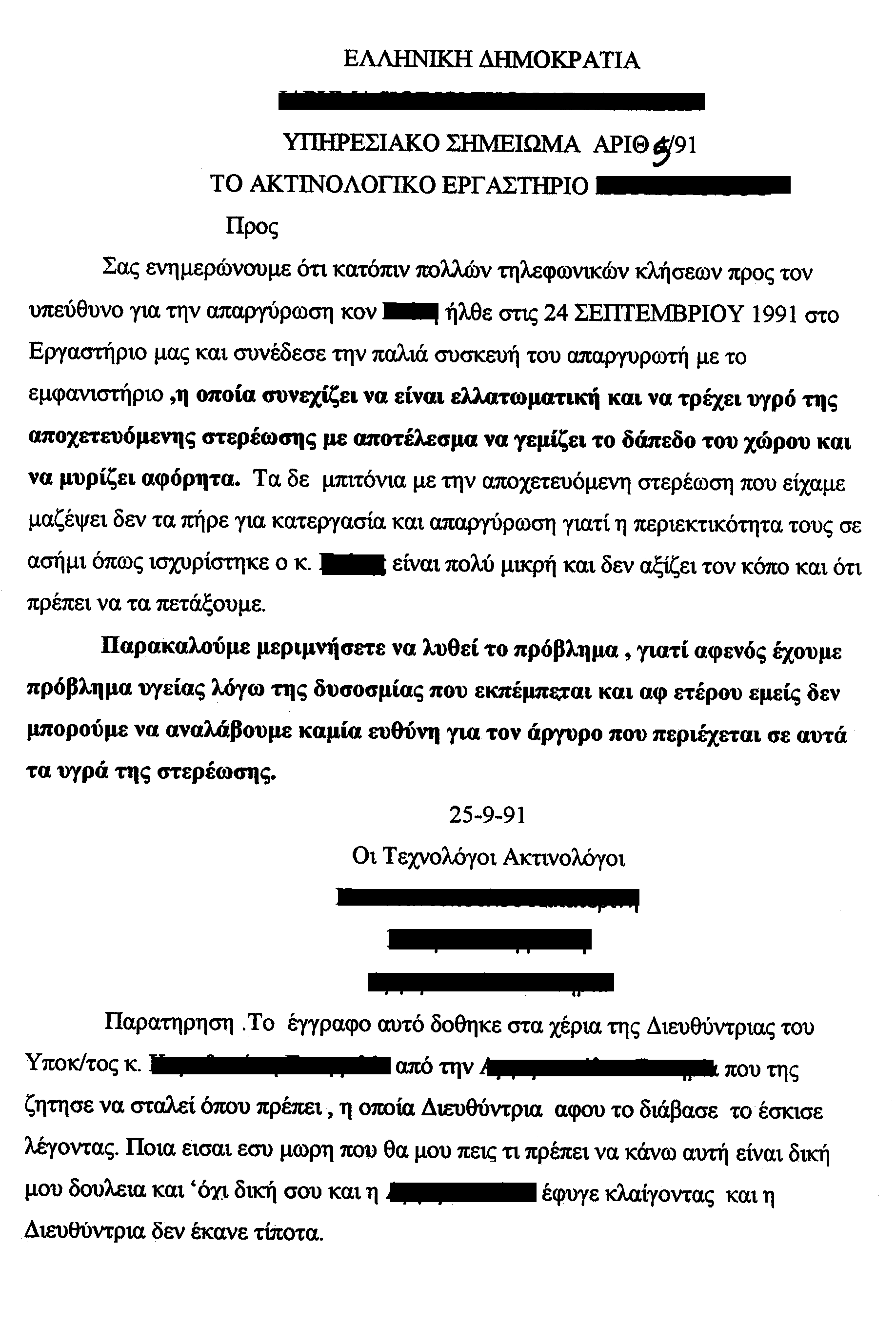 | 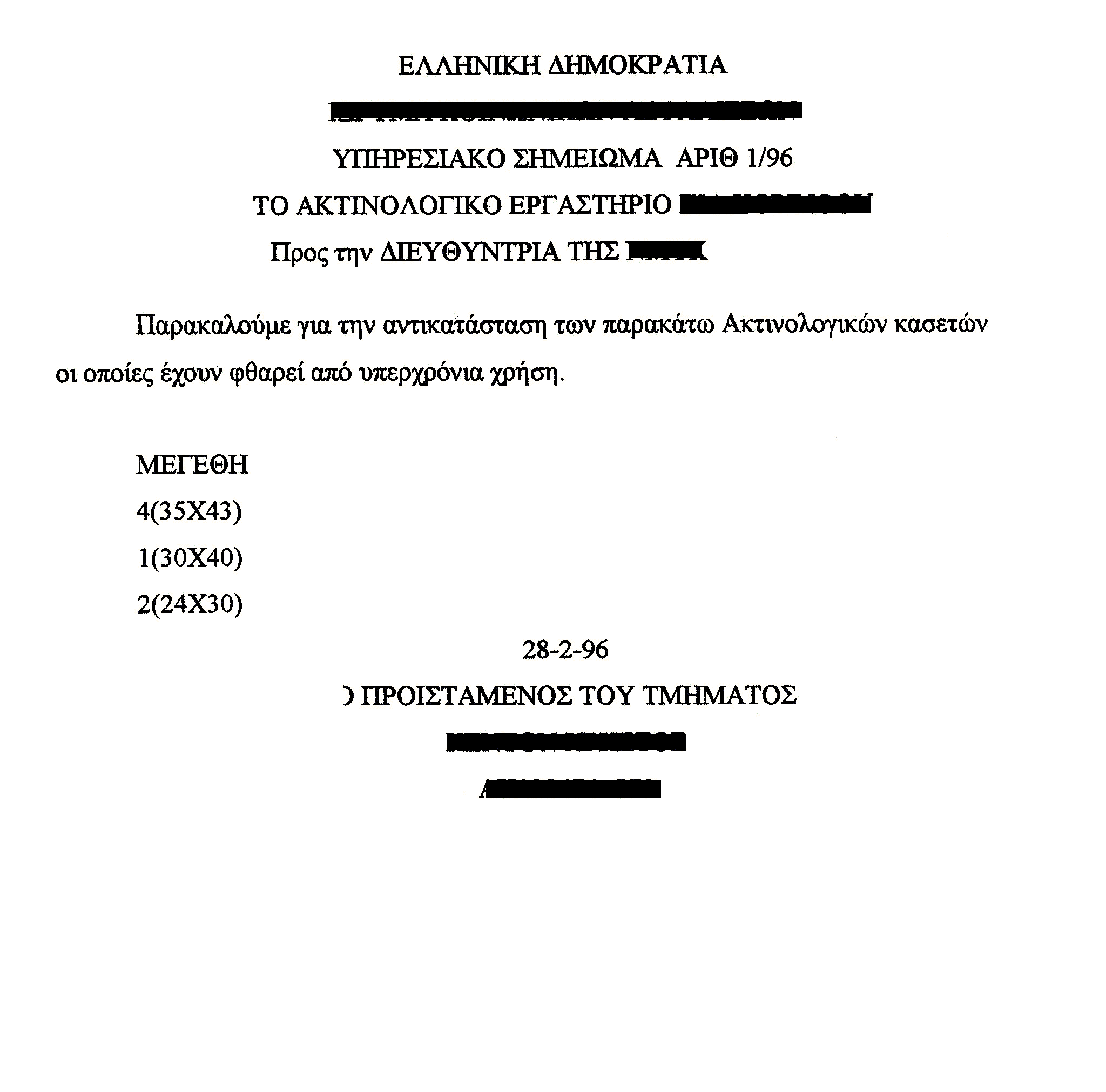 |
| --- | --- |

| 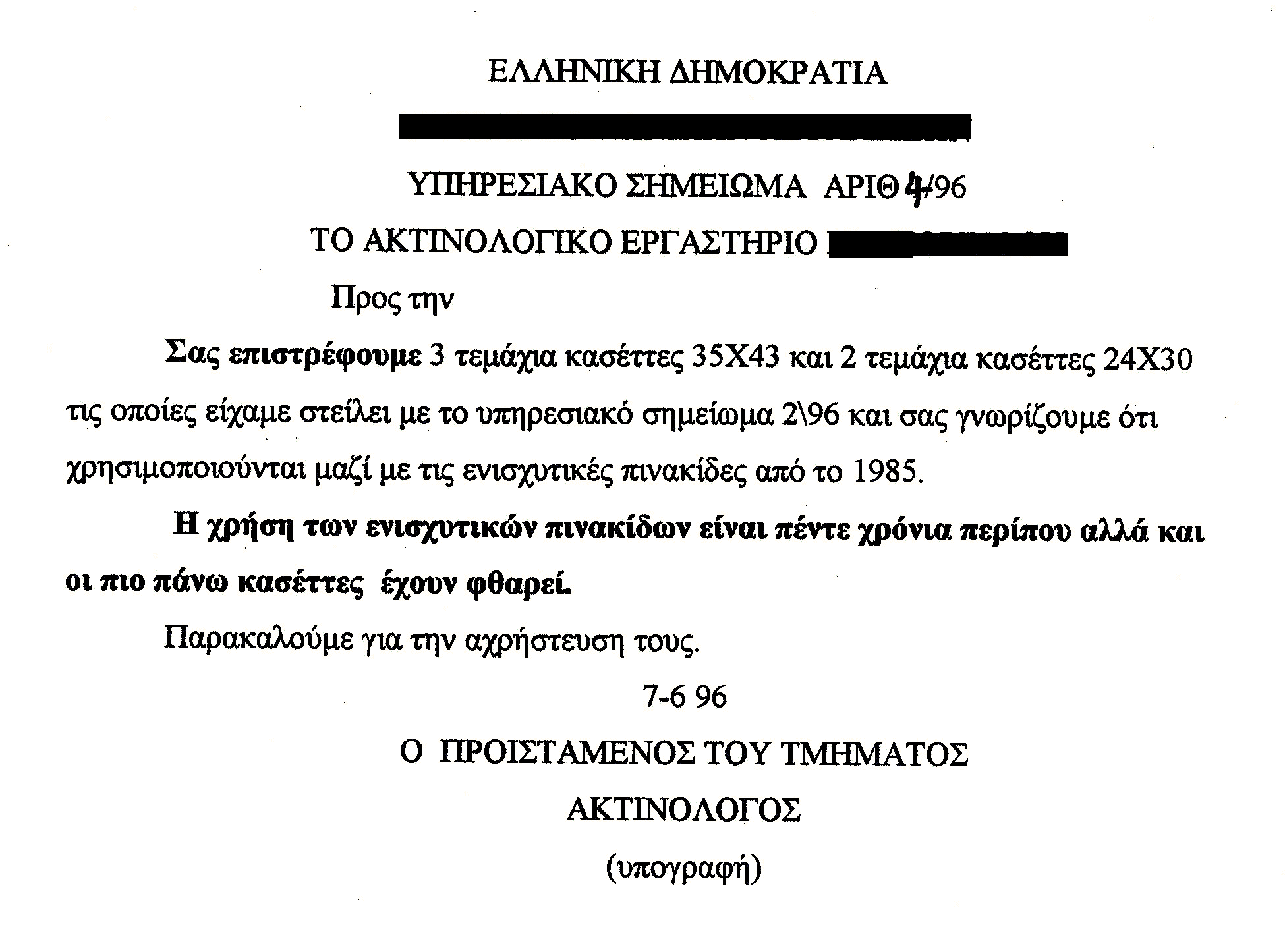 | 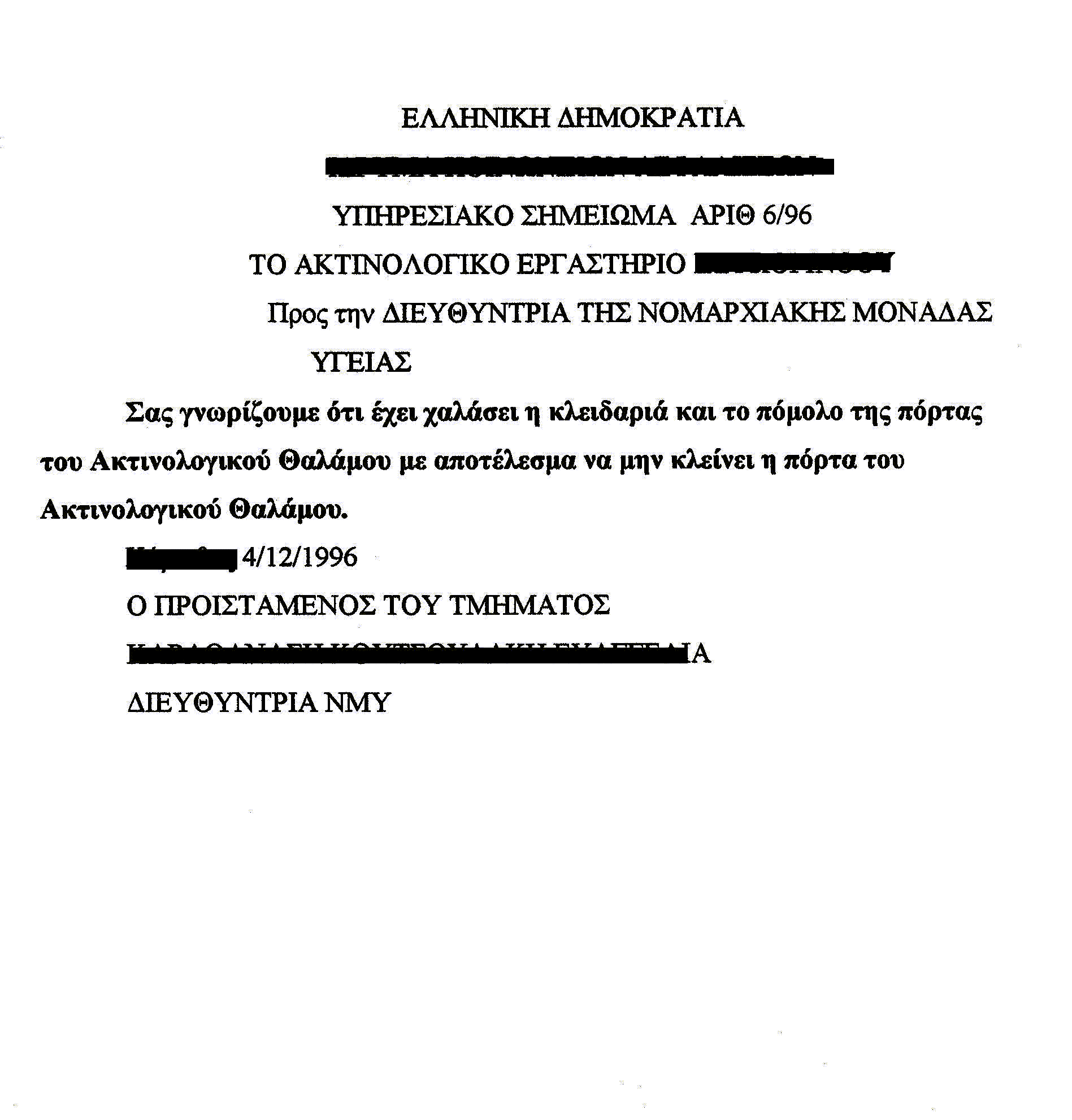 |
| --- | --- |

| 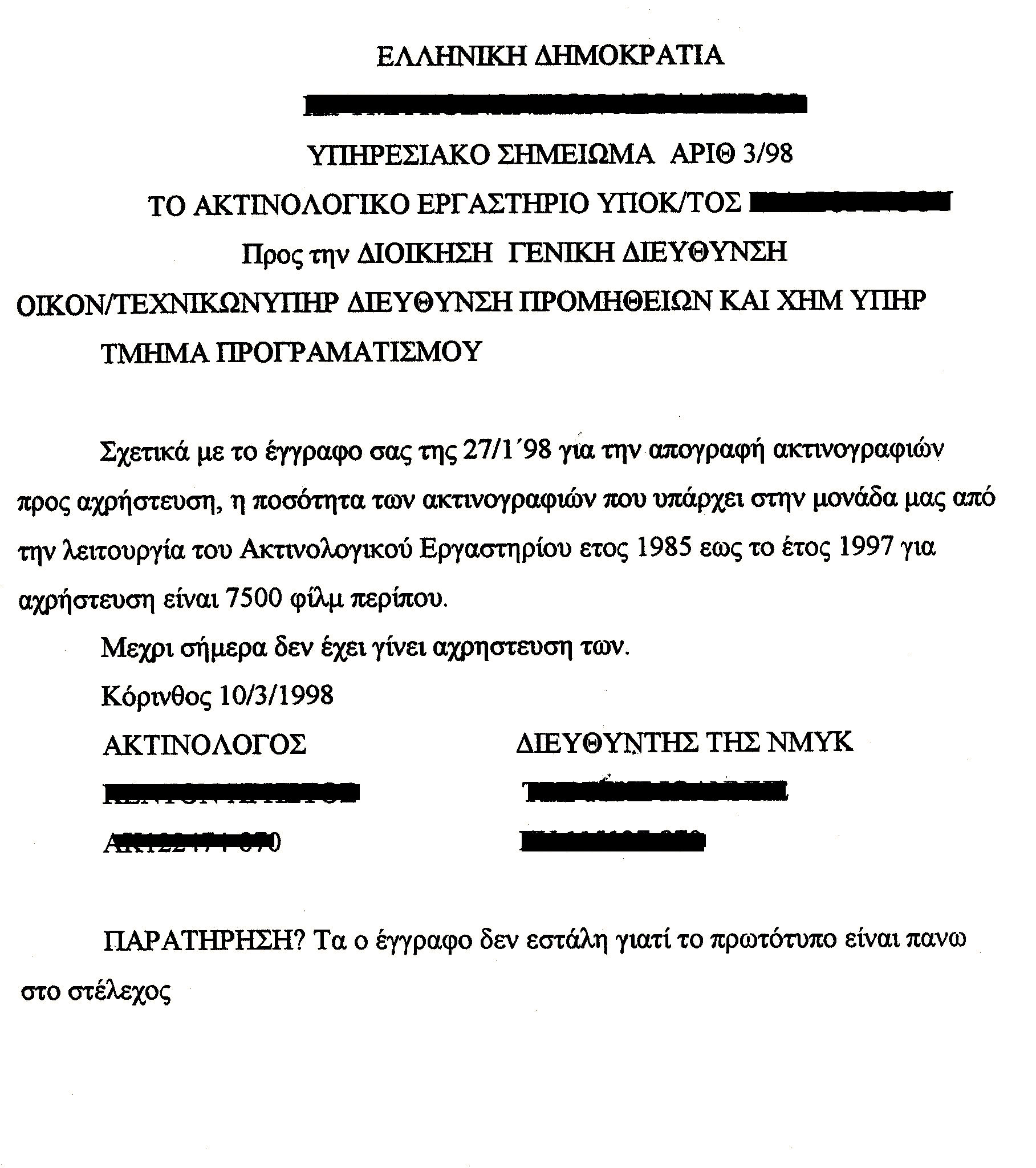 | 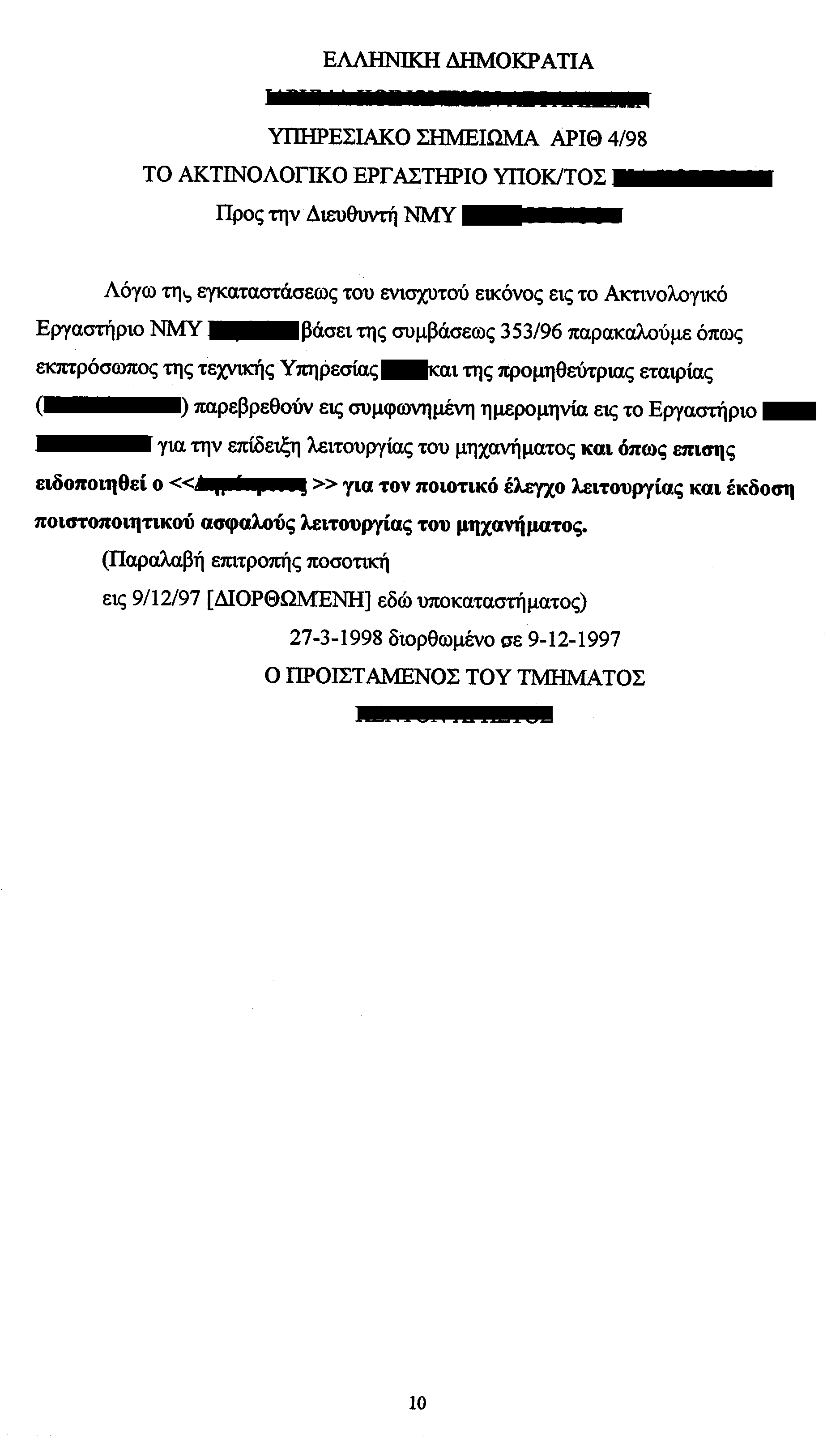 |
| --- | --- |

| 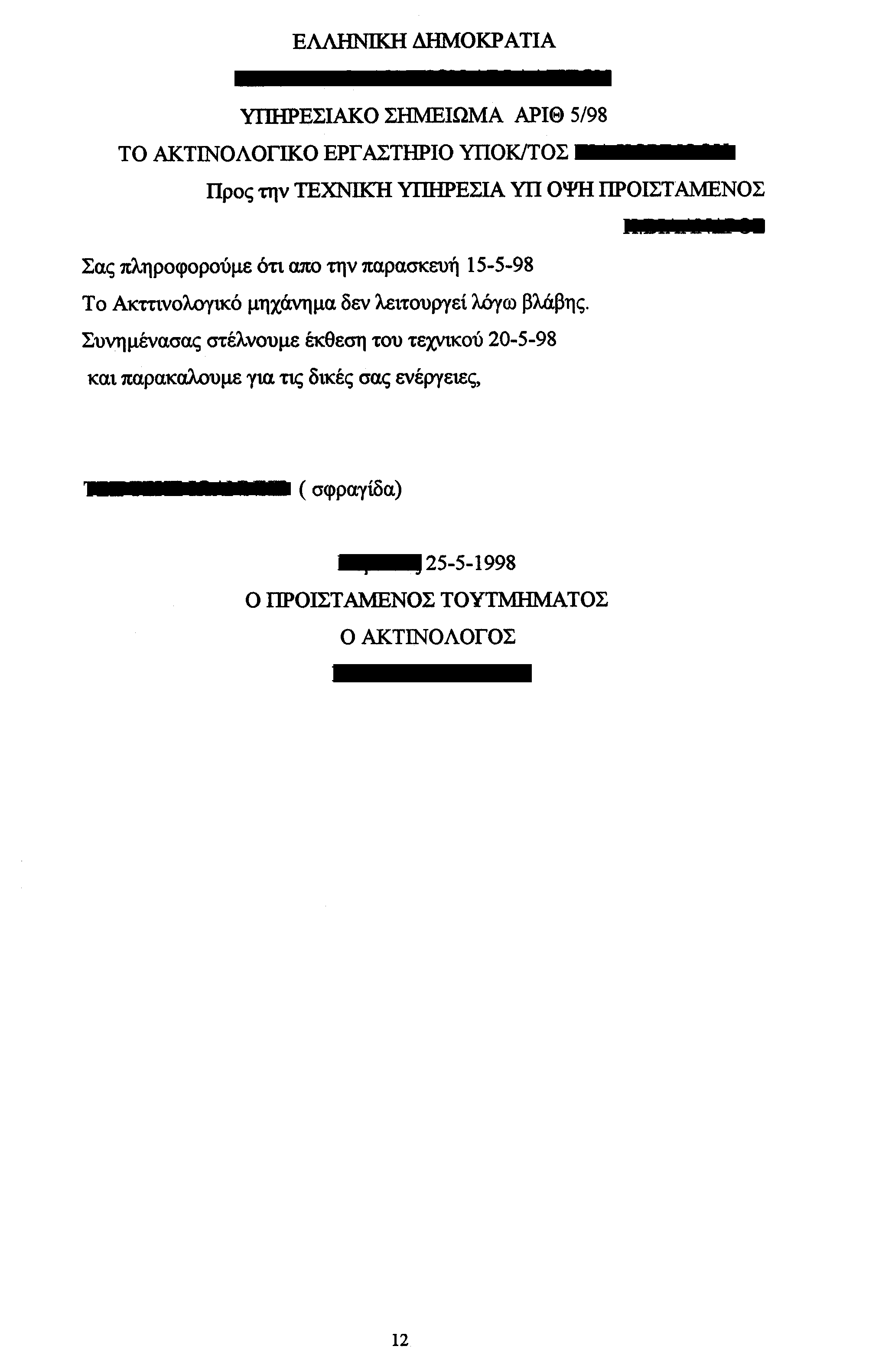 | 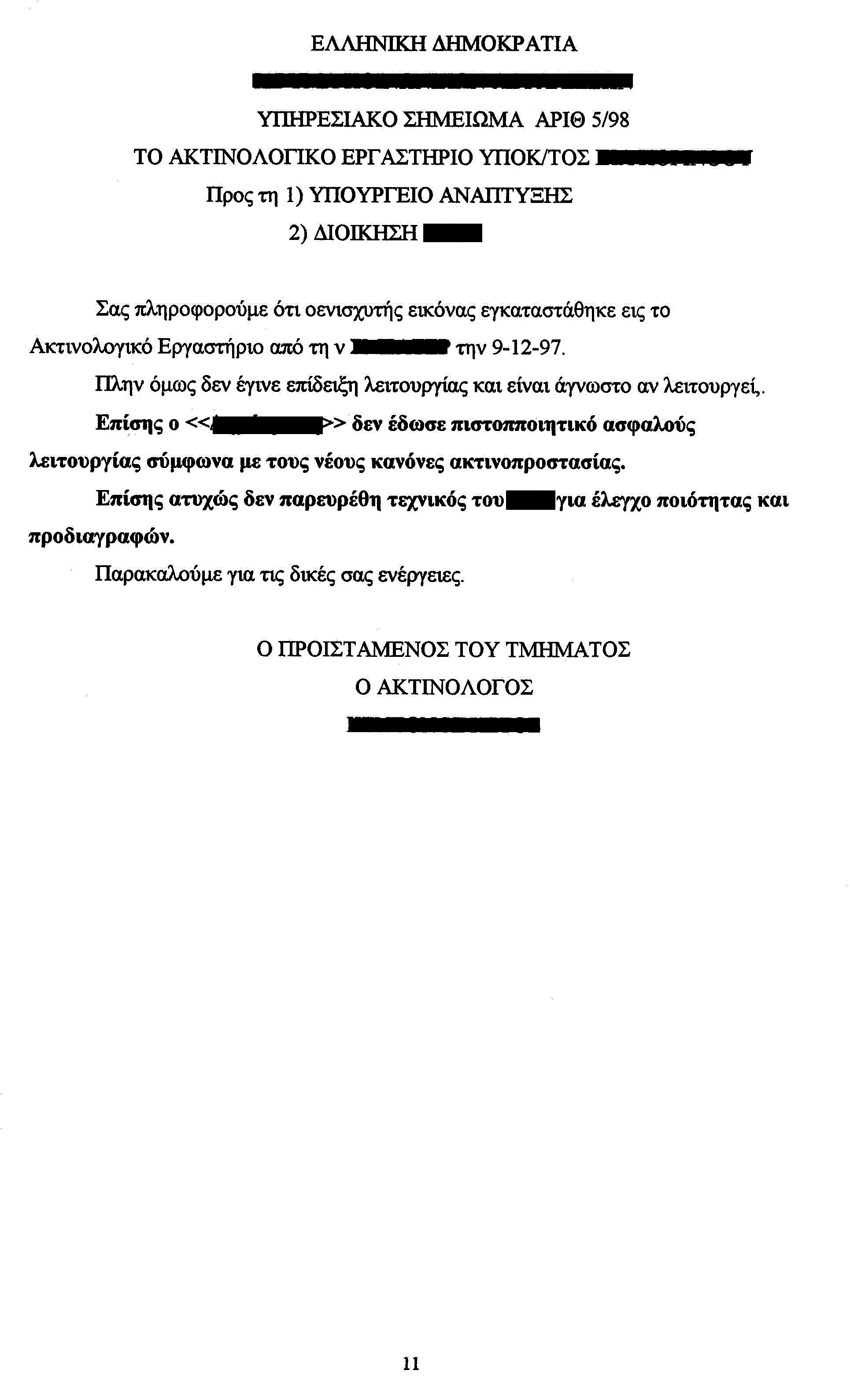 |
| --- | --- |

| 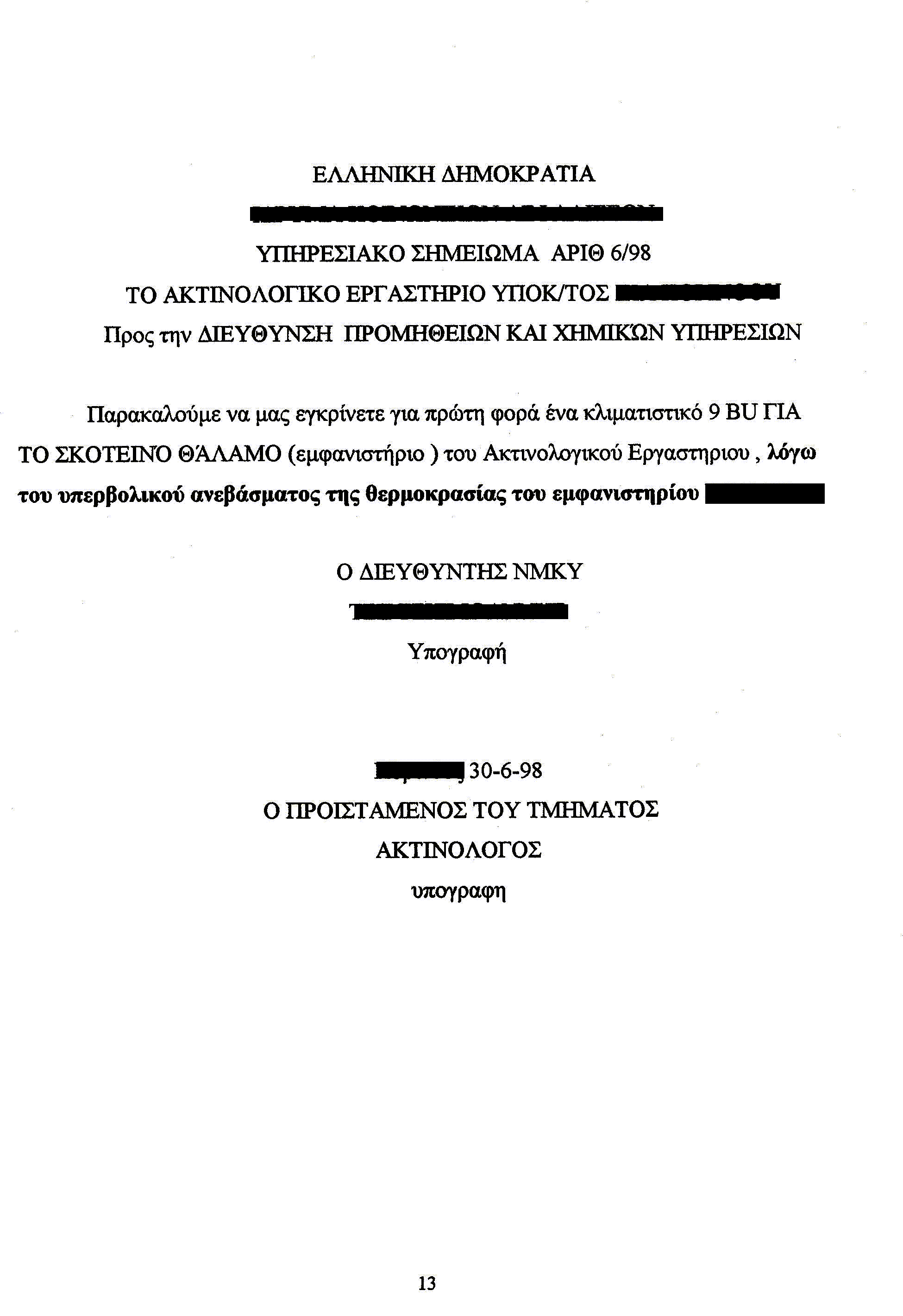 | 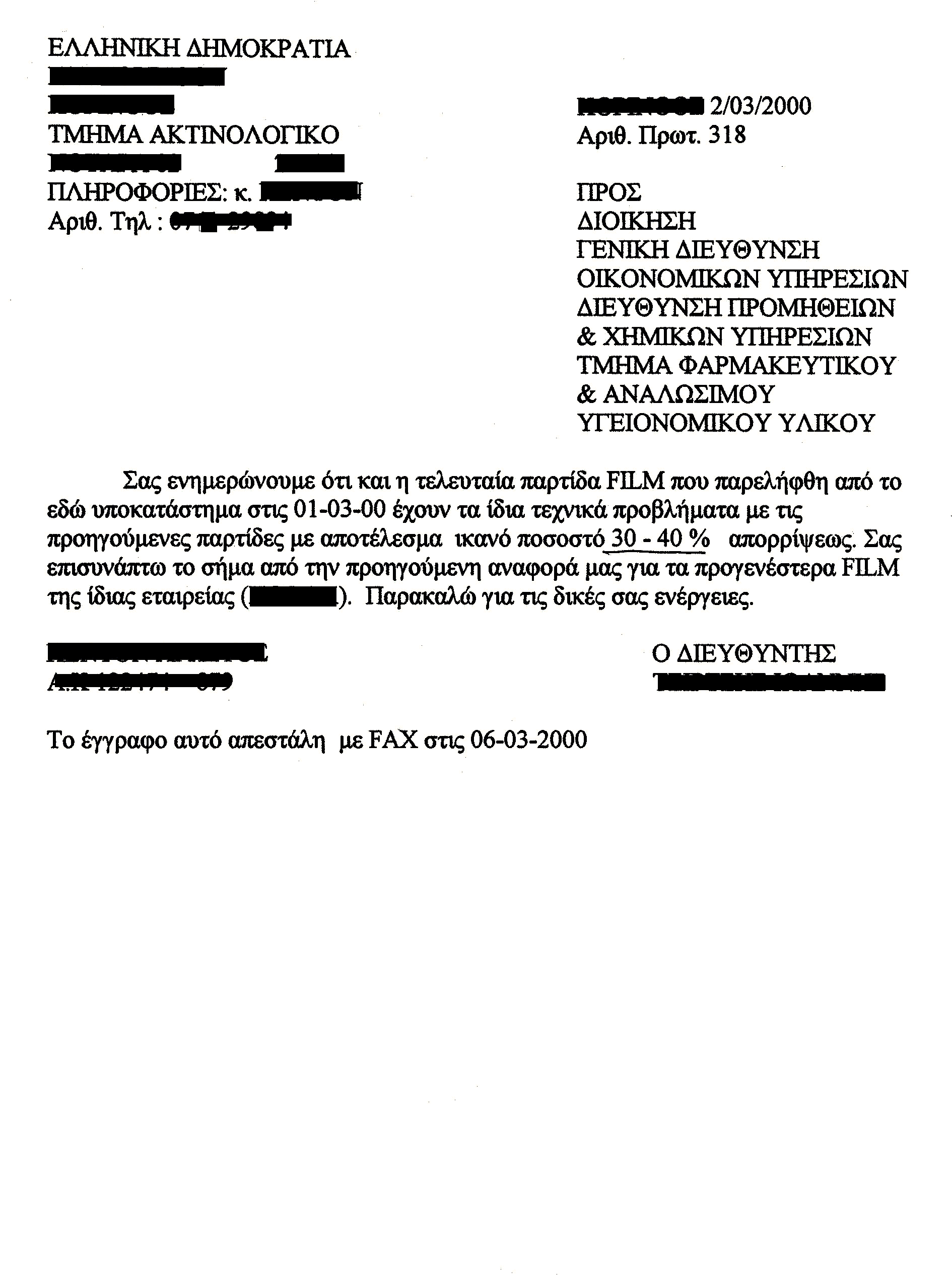 |
| --- | --- |

| 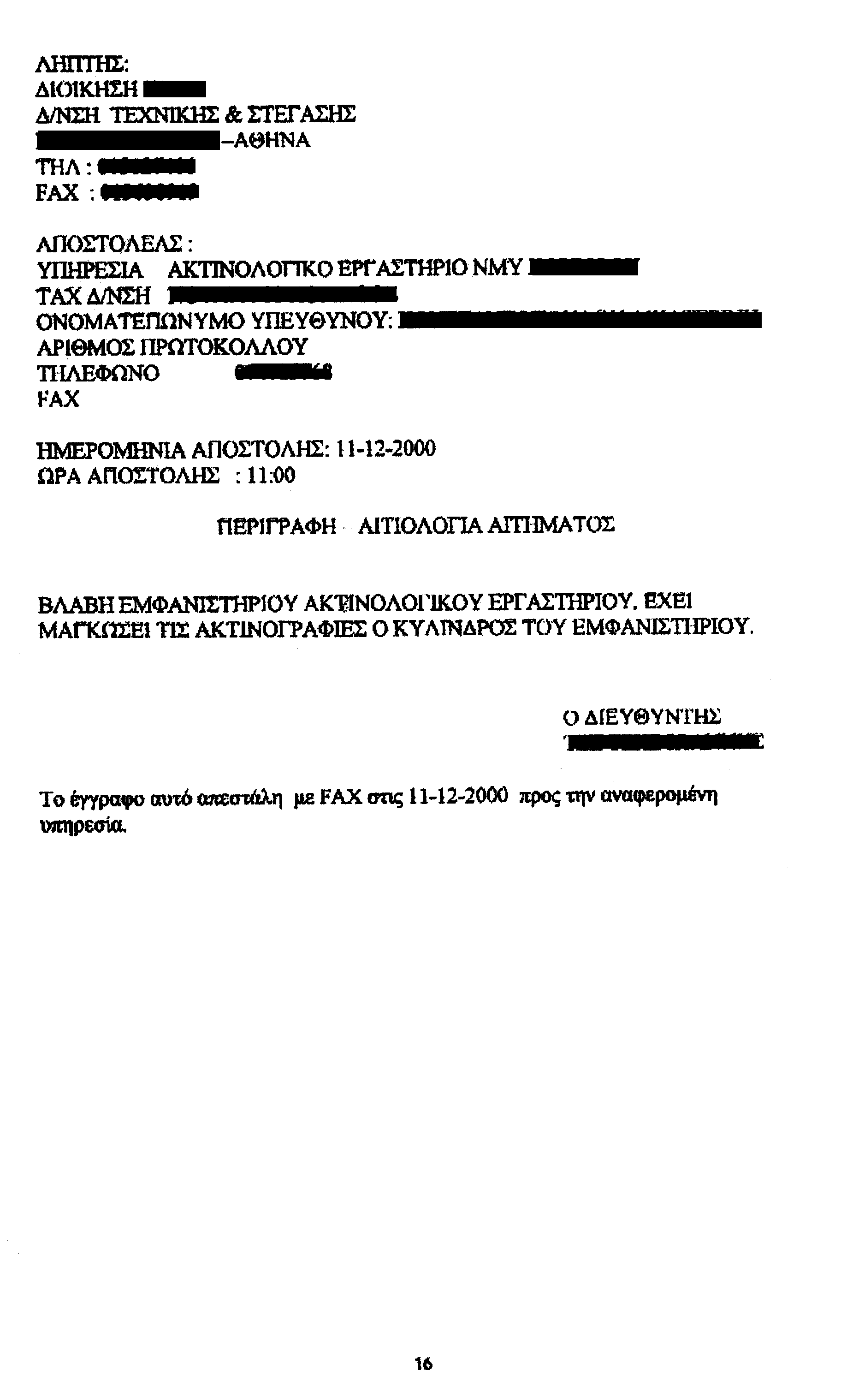 | 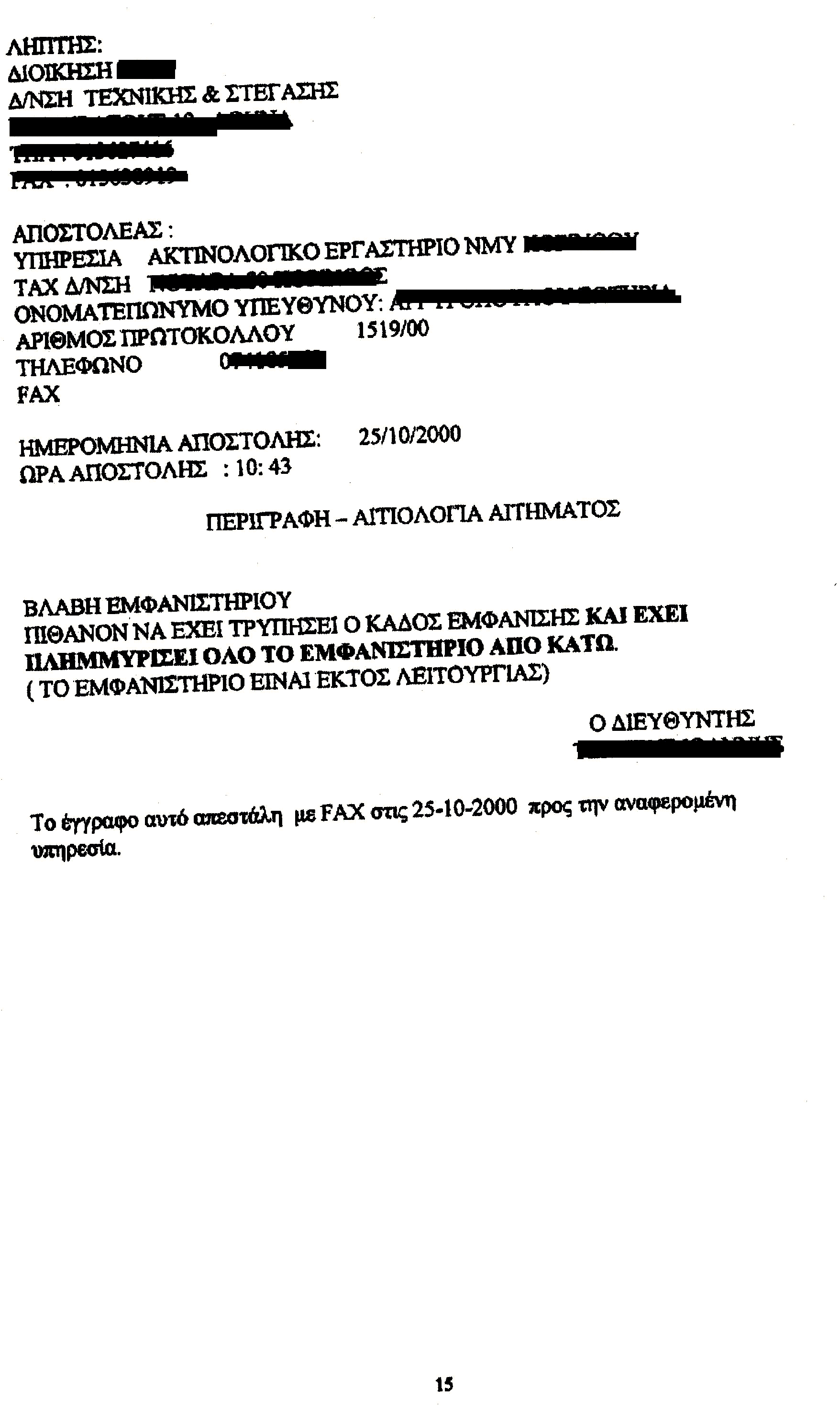 |
| --- | --- |

Supplement: Additional File 2 — Official administrative notes of employees to employer. The data provided represent the notes/requests of employees the period 1990–2000. [file 1745-6673-1-19-S2.doc]

| 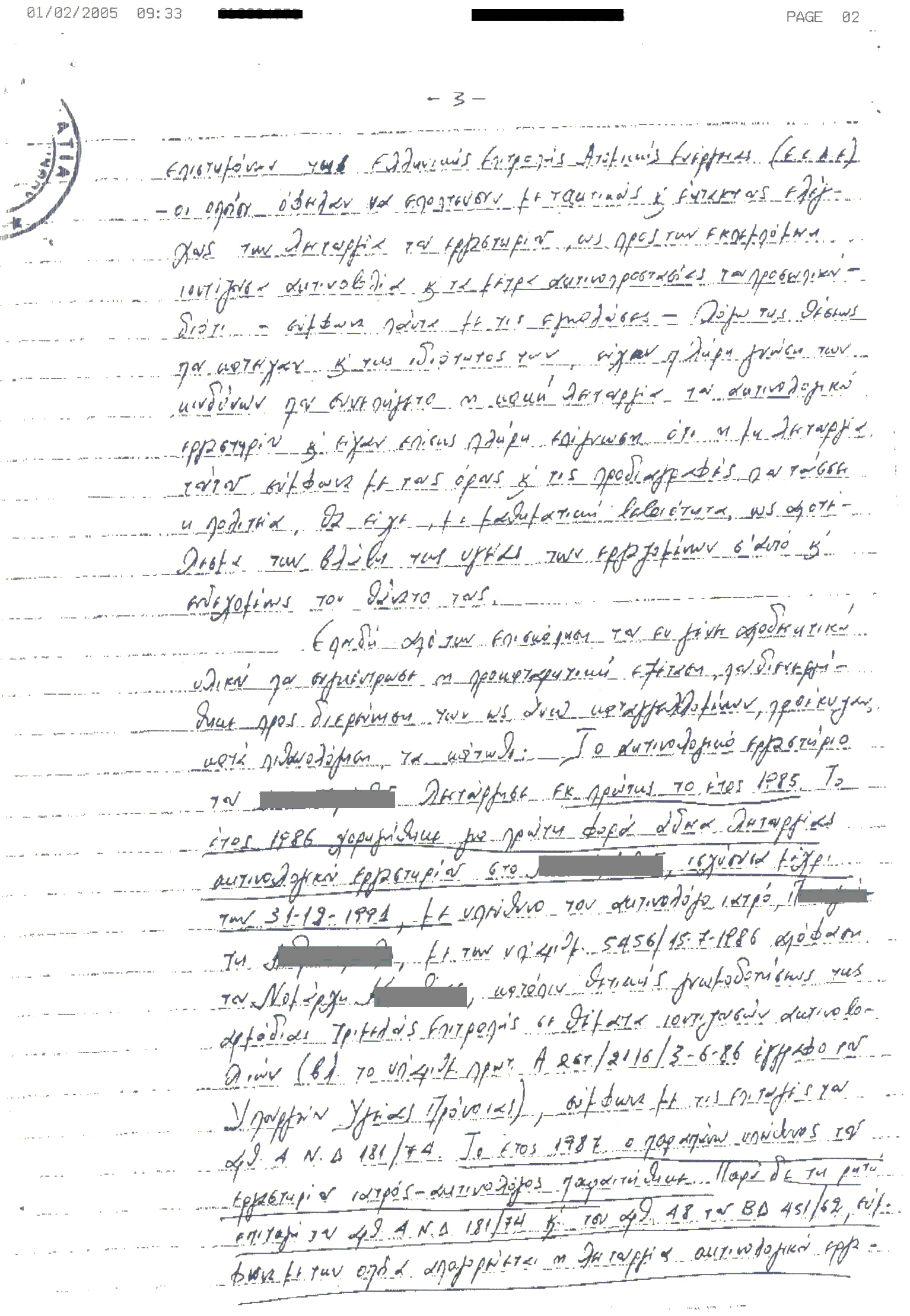 | 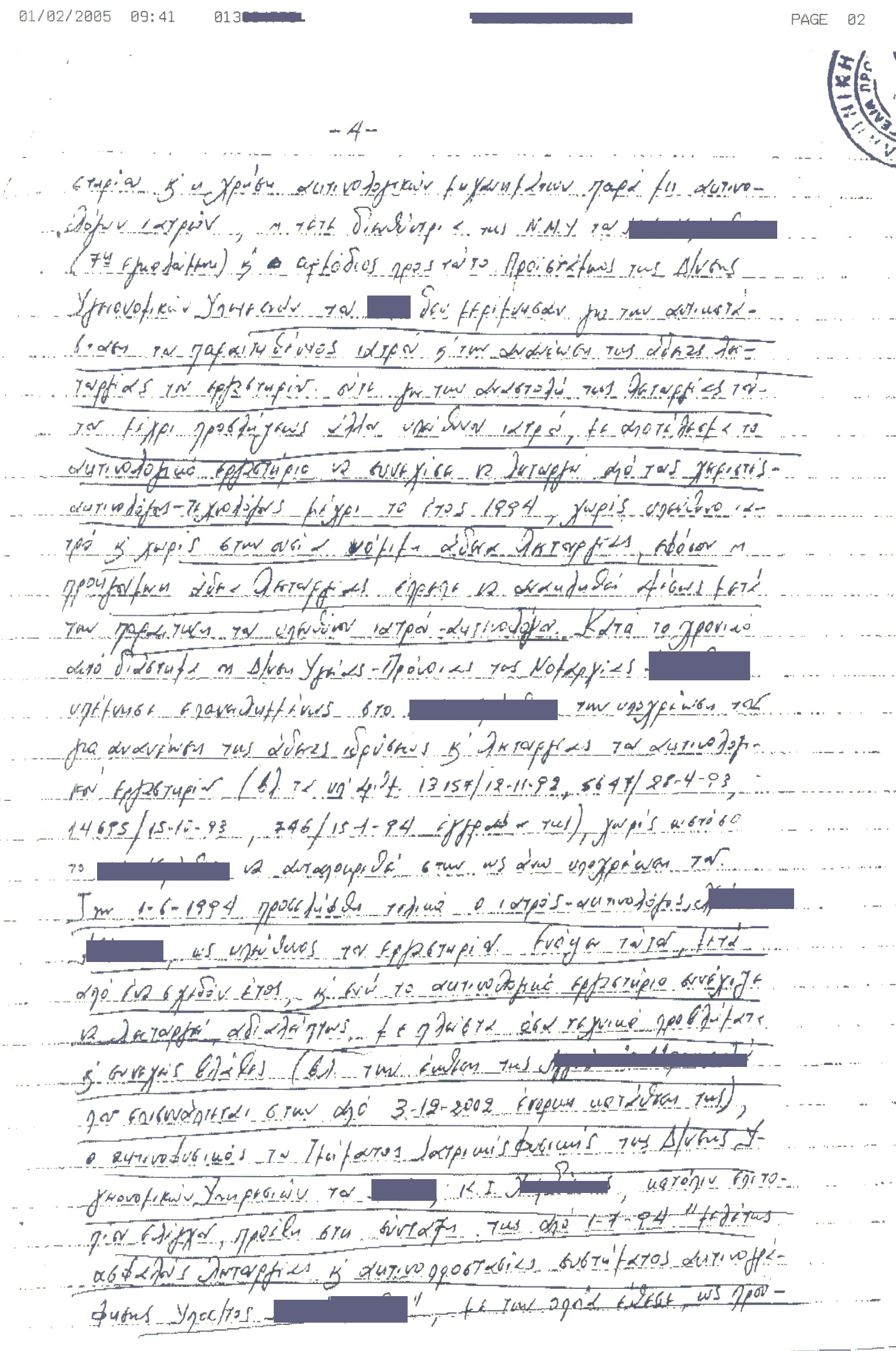 |
| --- | --- |

| 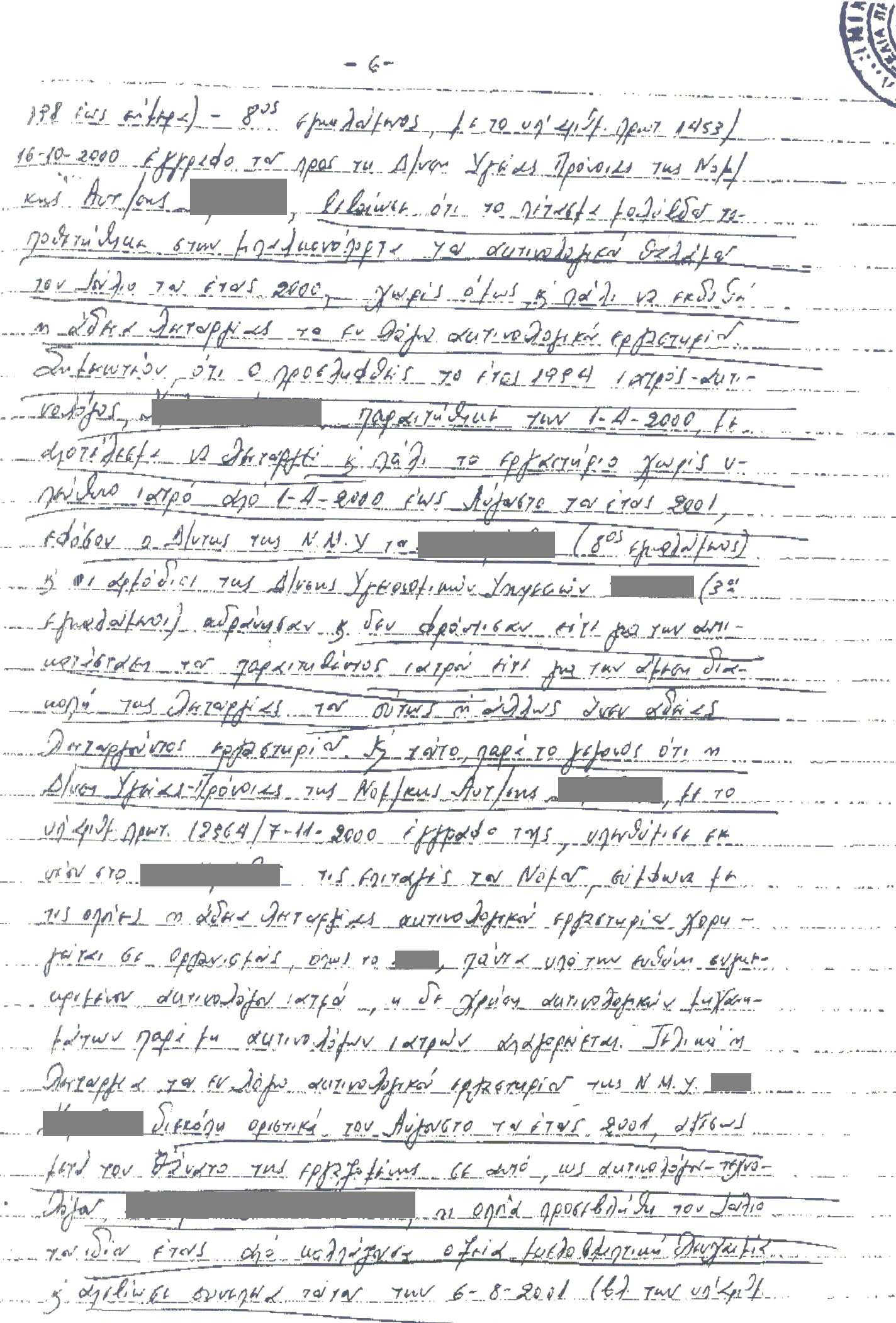 | 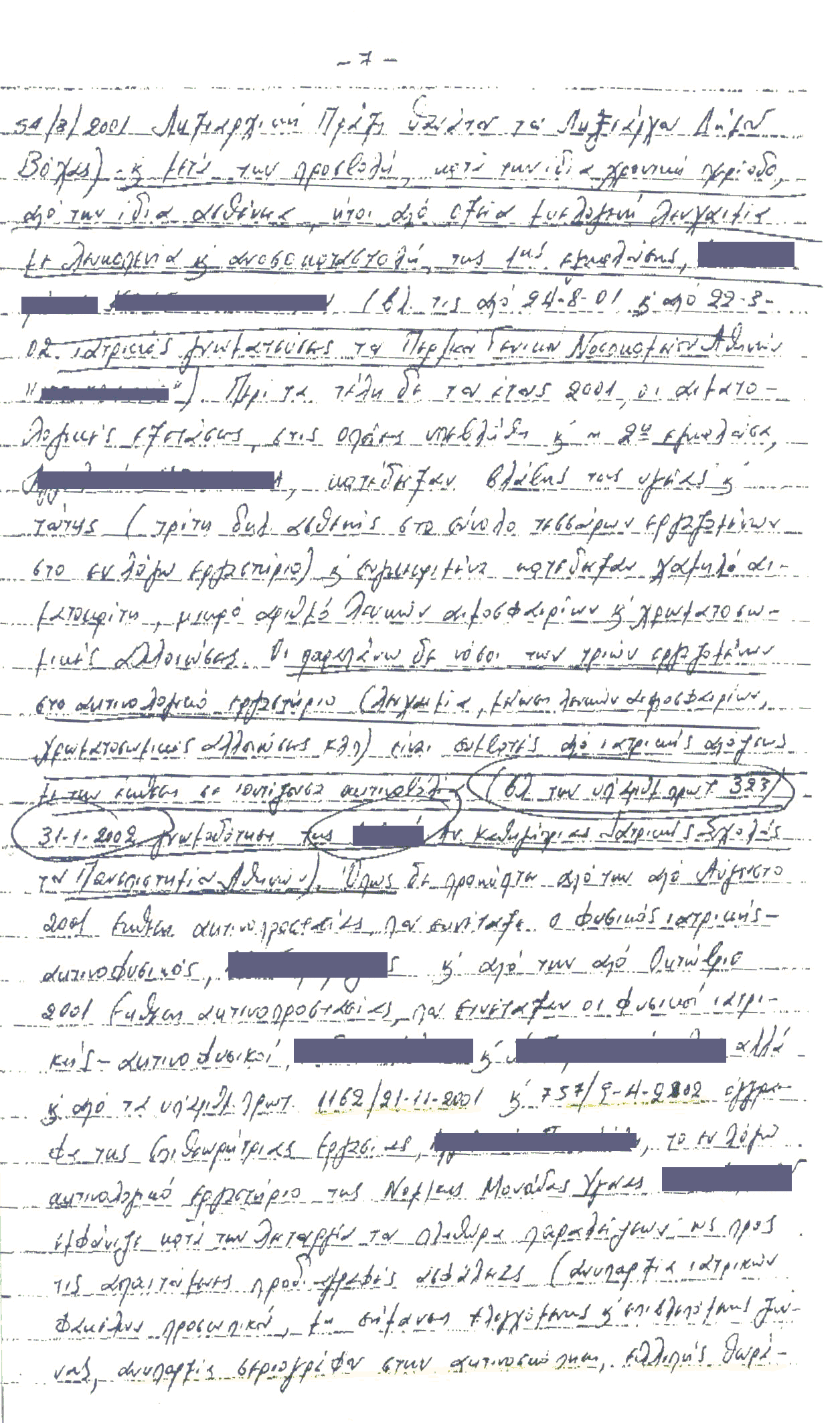 |
| --- | --- |

| 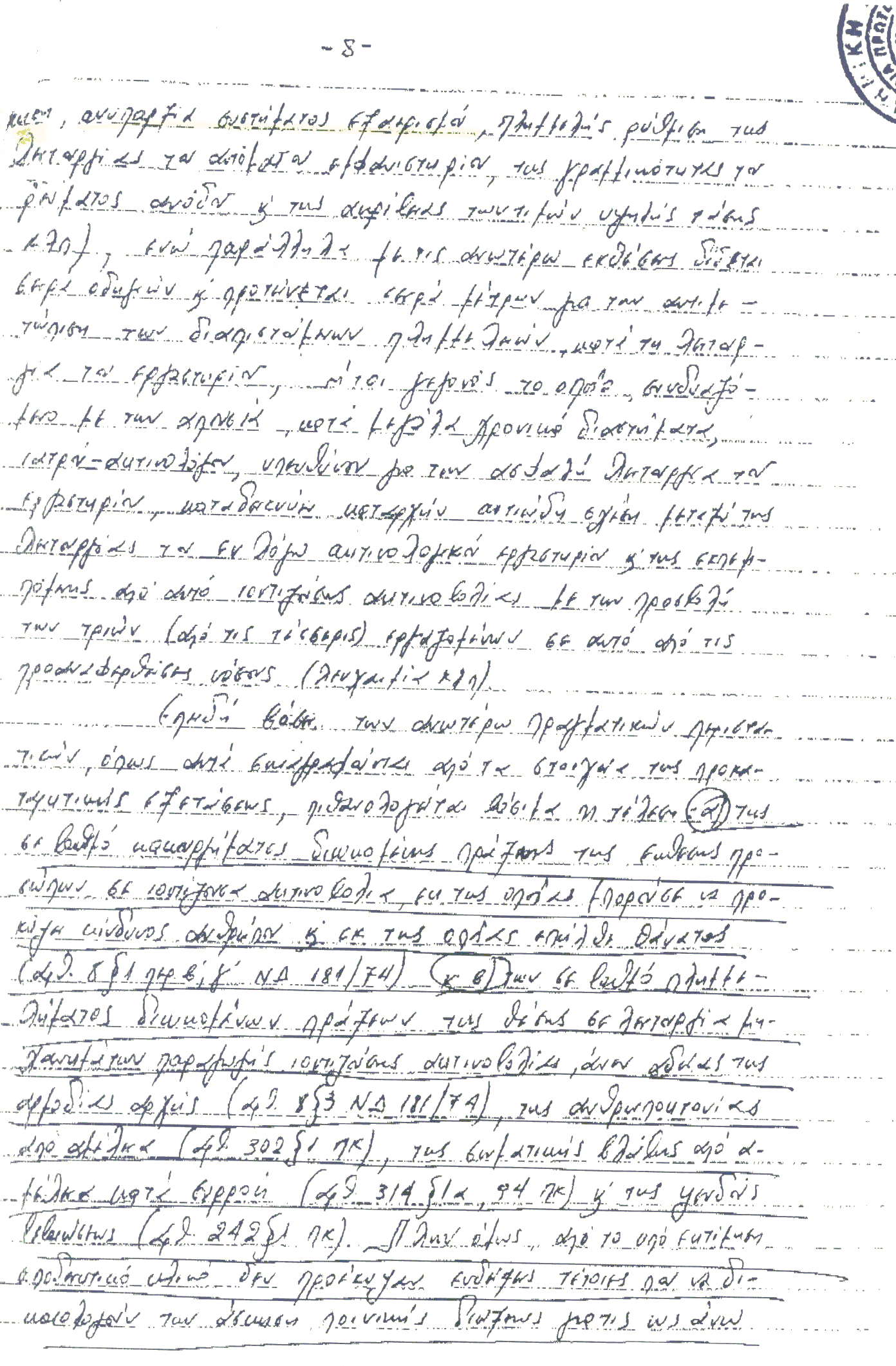 | 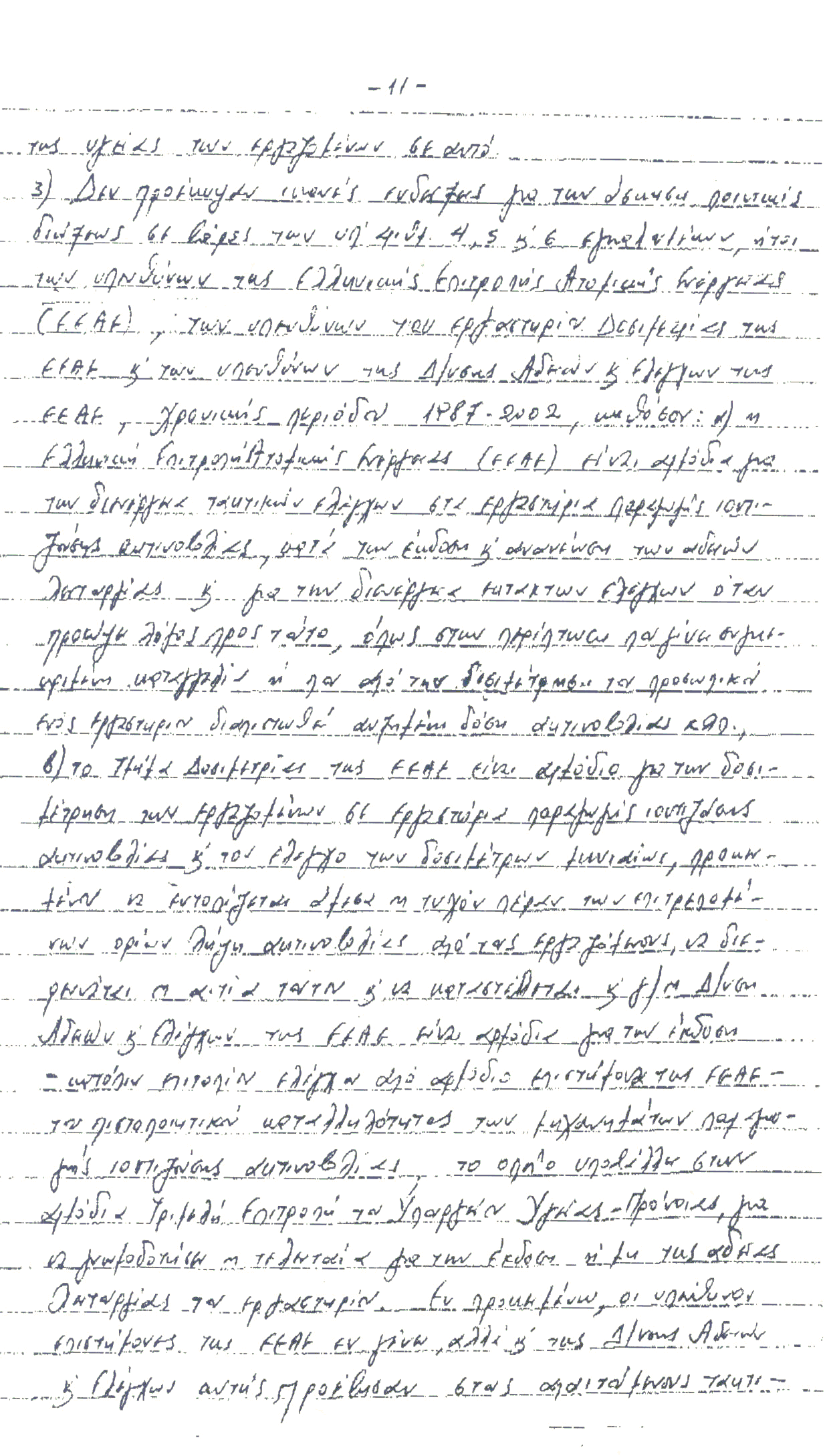 |
| --- | --- |

| 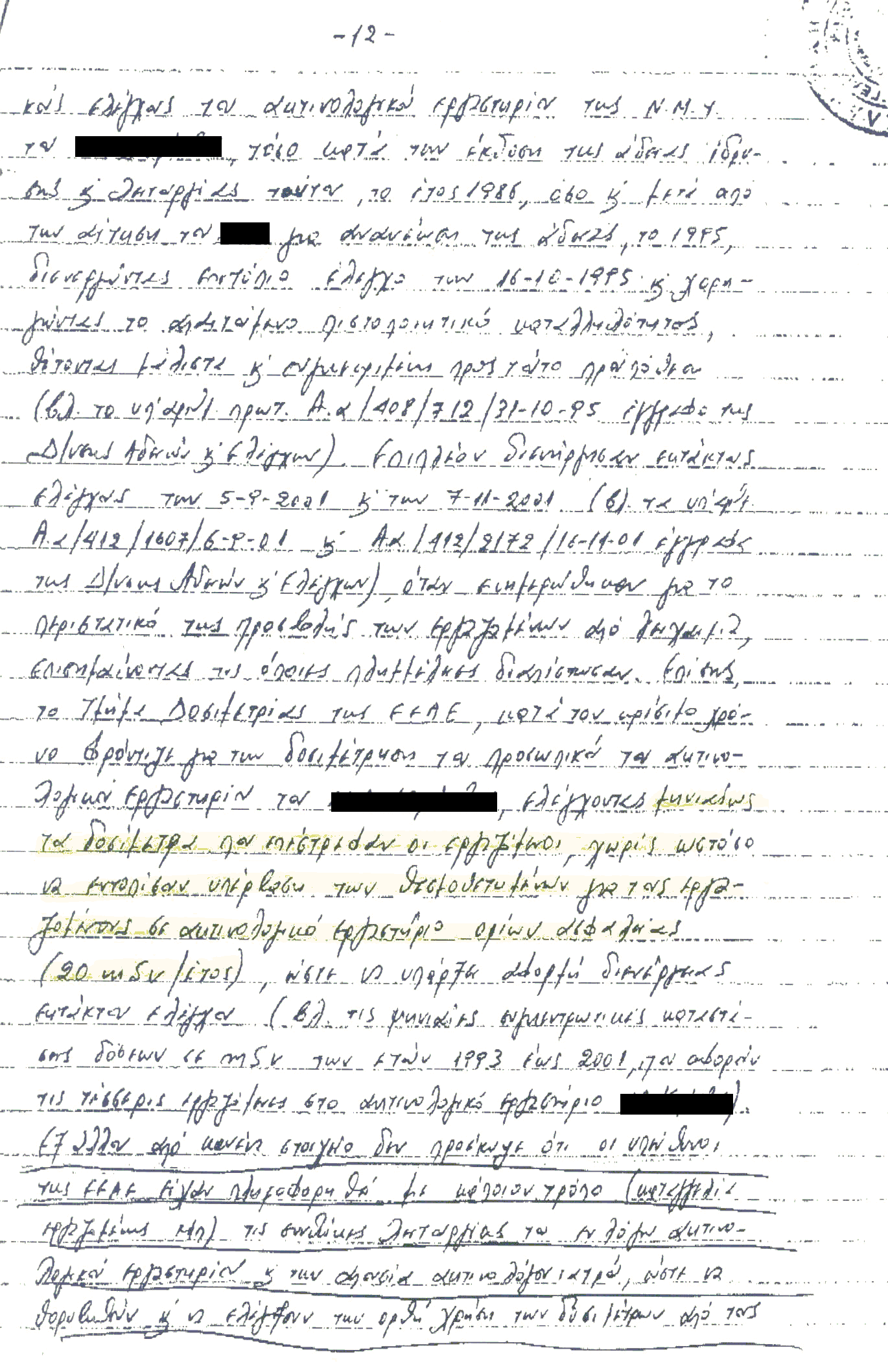 | 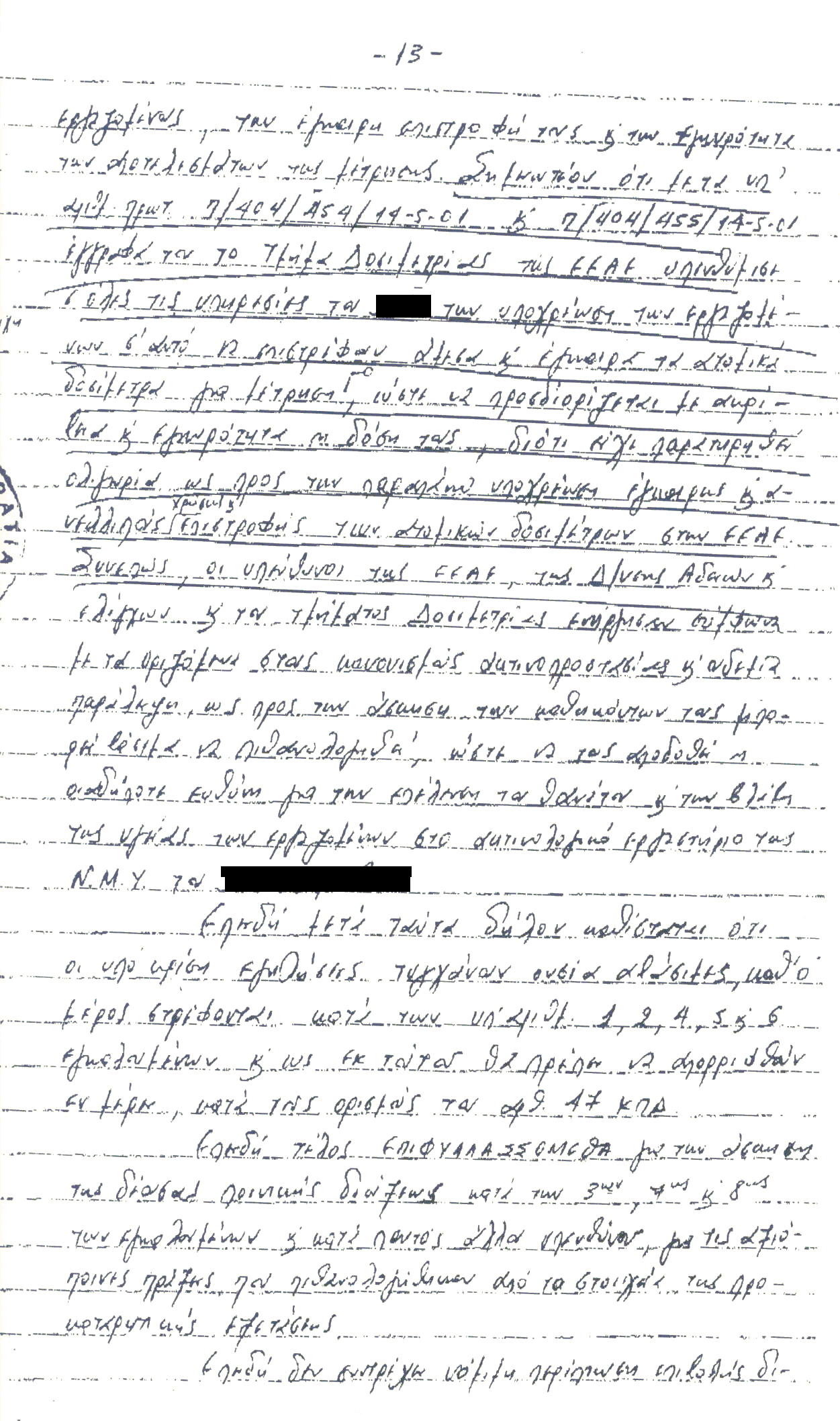 |
| --- | --- |

Supplement: Additional File 3 — District attorney's report. The data provided represent selected text from district attorney's report. [file 1745-6673-1-19-S3.doc]
